# Expanding structural diversity in a library of disulfide macrocycles through in-situ imide hydrolysis

**DOI:** 10.1038/s41598-021-03944-y

**Published:** 2022-01-07

**Authors:** Marcin Konopka, Artur R. Stefankiewicz

**Affiliations:** 1 Faculty of Chemistry, Adam Mickiewicz University, Uniwersytetu Poznańskiego 8, 61-614 Poznań, Poland; 2 Center for Advanced Technology, Adam Mickiewicz University, Uniwersytetu Poznańskiego 10, 61-614 Poznań, Poland

**Keywords:** Chemistry, Organic chemistry, Supramolecular chemistry, Chemical synthesis

## Abstract

We describe here an unorthodox approach to dynamic covalent chemistry in which the initially-unexpected in-situ hydrolysis of a bis-imide is employed to control the composition of a library of structurally diverse macrocycles. A single building block is used to generate a library of numerous disulfide-based architectures in a one-pot single-step process. The dual-stimuli method is based on simultaneous changes in pH and DMSO concentration to expand the structural diversity of the macrocyclic products. Mechanistic details of this complex process are investigated by the kinetics analysis. We delivered a facile strategy for the synthesis of water-soluble, multicomponent and dynamic macrocycles equipped with number of different functional groups, thus giving a prospect of their application in guest-driven phase transfer.

## Introduction

Dynamic combinatorial chemistry (DCC) is of continuing interest among various fields of modern chemistry^1–3^. Many reversible linkages are employed in DCC, such as imines, acyl hydrazones, disulfides, boronic esters, or coordination bonds^4–6^. Dynamic disulfides are of particular interest due to their useful applications as chemical receptors^7,8^, nanocapsules for molecular transport^9–11^, or in metallosupramolecular architectures^12^. Recently, there has been a growing interest in the search for new methods and conditions for the oxidation of thiols to disulfides in DCC using metalloid catalysis^13,14^, solvent effects^15^, iodine^16^ or mechanochemical stimuli^17–19^. To date, numerous water-soluble disulfide systems of distinct topologies such as macrocycles, cages, catenanes or knots have been described, most of which are based on organic cysteine-functionalized components^20–29^. Macrocyclic structures, especially those based on modified naphthalenediimides (NDIs) have been heavily explored in the last decade as they present interesting properties and functions such as molecular sensors, host−guest complexes, molecular devices, catalysis through π-anion interactions and non-covalent binding with DNA for bio-applications^30,31^. Several different strategies of controlled, stimuli-based, modification of the dynamic library composition through physical and chemical factors have been described for these systems^32^. However, it remains a challenge to externally control DCL compositions and product constitutions.

**Figure 1:**
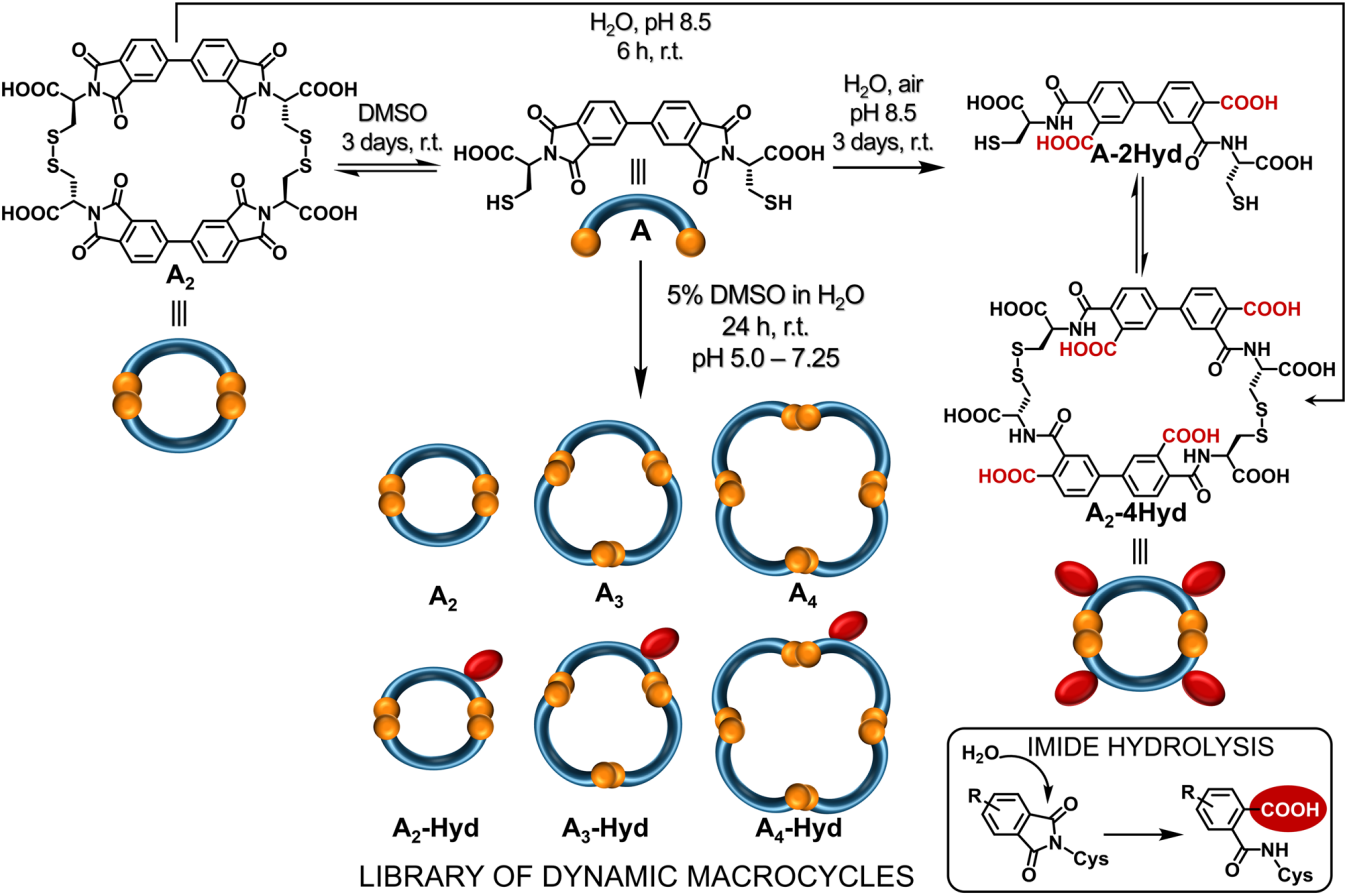
Schematic representation of the DCLs formed from component **A** under three different conditions, in DMSO, in water at pH 8.5, and in 5% DMSO/aqueous AcONH_4_ buffer. The inset contains a diagram of the irreversible imide hydrolysis. The –COOH group resulting from the imide hydrolysis is marked as a red ellipse.

Here we present an unorthodox approach to disulfide DCC by employing *in-situ* imide hydrolysis which leads in our system to expanded library diversity and larger macrocycles. We describe a robust method for the use of a mixture of water and DMSO as an excellent environment for the oxidation and exchange of thiols and we show the control of the library composition through appropriate pH and DMSO concentration. Previously, we described new building blocks based on 3,3′,4,4′*-*Biphenyltetracarboxylic dianhydride (BPDA)^33^, similar to the NDI-based components, but with an extra degree of geometrical flexibility. One of these was the bis-imide equipped with two cysteine moieties **A** (Fig. 1). During the standard DCL procedure (water, pH 8.0) with **A**, instead of the expected macrocyclic oligomer library we noticed products resulting from the hydrolysis of **A**. Literature evidence on this process encouraged us to investigate the potential of this phenomenon in concert with disulfide DCC^34,35^. In basic aqueous solution, imide hydrolysis may take place, and results in opening of the five-membered ring, thereby providing additional features in conformational flexibility, polarity (new carboxylic groups) and intramolecular non-covalent interactions (NH hydrogen bond donor) during disulfide formation.

## Results and disussion

The work began with the synthesis of dithiol component **A** starting from the BPDA and S-Trityl-L-cysteine. We employed our earlier reported microwave method^33^ to obtain an intermediate STr-protected diimide product. In the second step the trityl groups were removed by the standard Et_3_SiH/TFA/DCM reaction. Natural L-cysteine provides several important functions for further self-assembly, such as the carboxyl groups ensuring high polarity and water solubility, a stable amino-acid chiral centre, and -SH groups for oxidation located on a conformationally labile arm.

Additionally, the component **A** contains a single bond between two phenyl rings, which provides additional rotational flexibility to the entire system, a significant difference compared with rigid NDIs.

**Figure 2:**
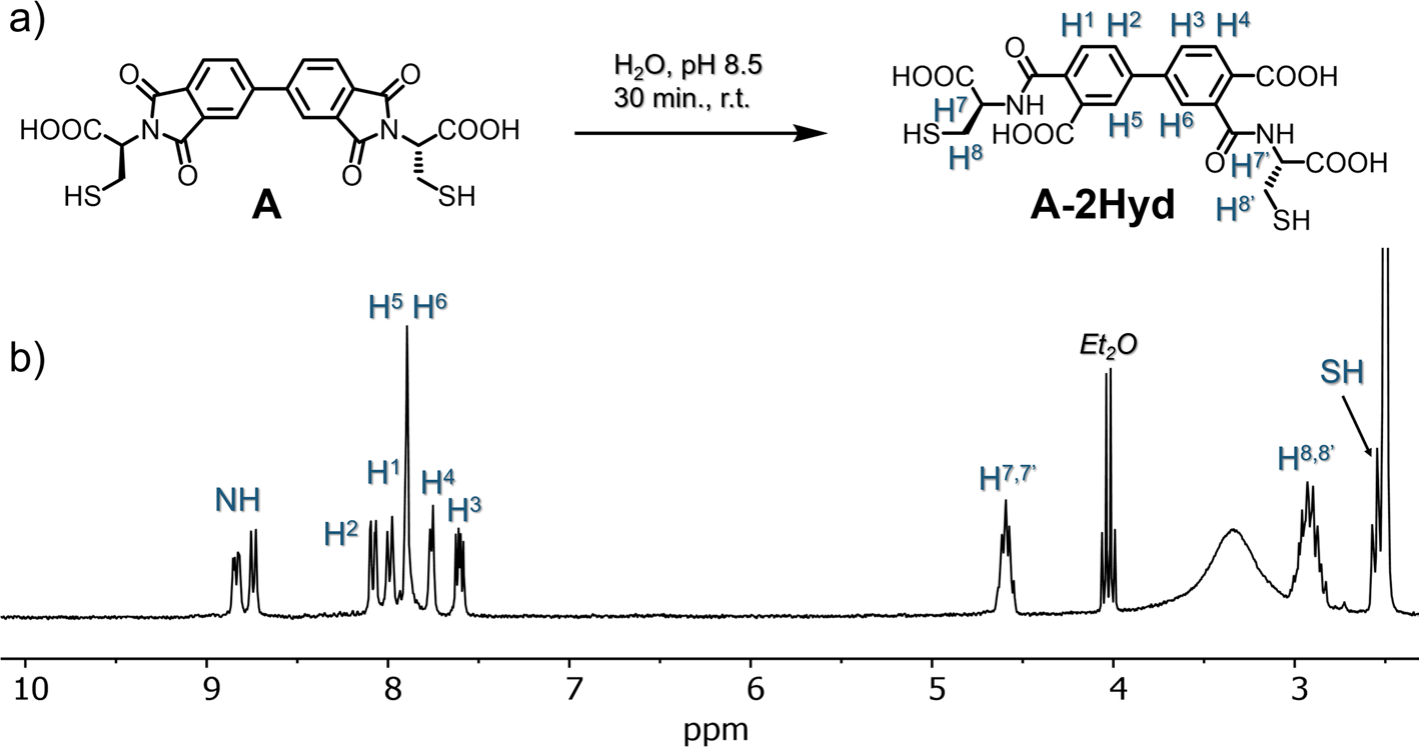
(**a**) Scheme of the unsymmetric hydrolysis of **A** into **A-2Hyd**, (**b**) ^1^H NMR spectrum (300 MHz, 298 K, DMSO-d_6_) of **A-2Hyd**.

In the first experiment a simple aqueous solution of **A** at pH 8.5 in unsealed vial was set up (see ESI S2 for details). After several minutes we observed complete decay of **A** and the formation of a single new product. This was isolated and based on NMR and MS analysis was shown to be the unsymmetrical product of double hydrolysis of **A** where the newly formed two amide groups are placed *para* and *meta* to the inter-biphenyl bond **A-2Hyd** (Fig. 2). Analysis of the ^1^H and COSY (see Fig. S8 in ESI) spectra in DMSO clearly shows the formation of a pair of amide N–H signals (δ8.83 and δ8.75 ppm) coupled to a pair of methine C-H cysteine signals.

**Figure 3:**
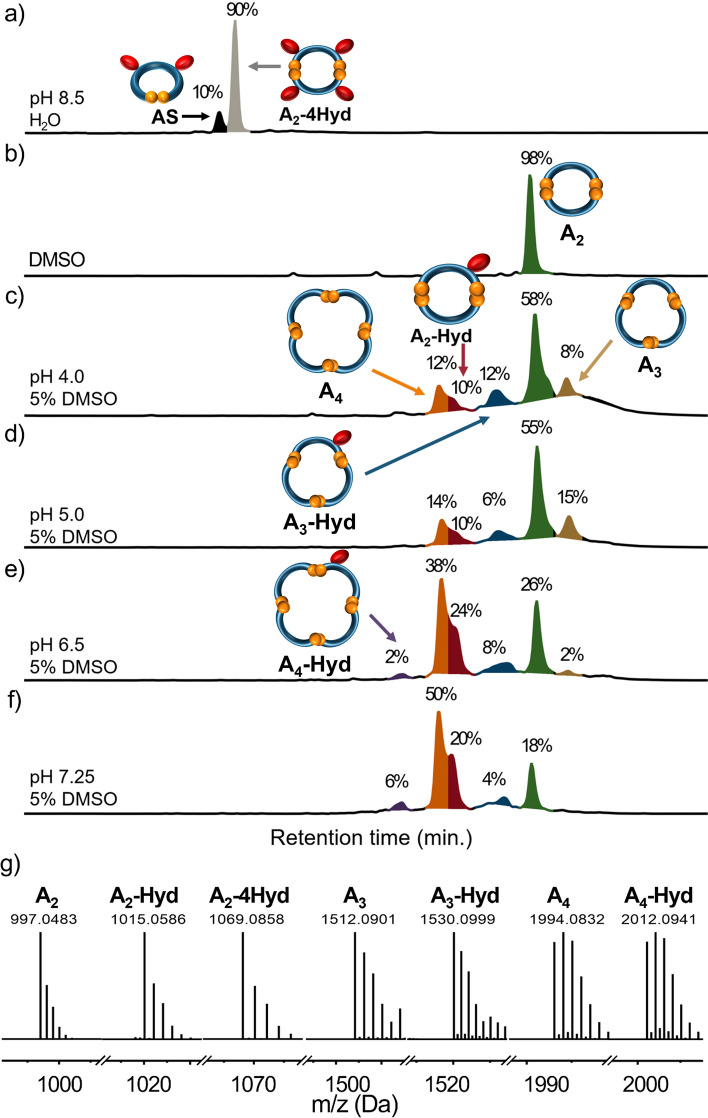
(**a**–**f**) HPLC chromatograms (254 nm) of 3 days old **A** DCLs in various conditions of DMSO concentration and pH. The library material distribution is represented as percentages next to the corresponding peaks (relative peak area). The peak colour indicates each macrocycle. See Figs. S17–S24 for LC–MS details. (**g**) HR-MS analysis of macrocycles as ions [M + H]^+^ (or [M + NH_4_]^+^ for **A**_**3**_, **A**_**3**_**-Hyd**, see Figs. S18–27).

**Figure 4:**
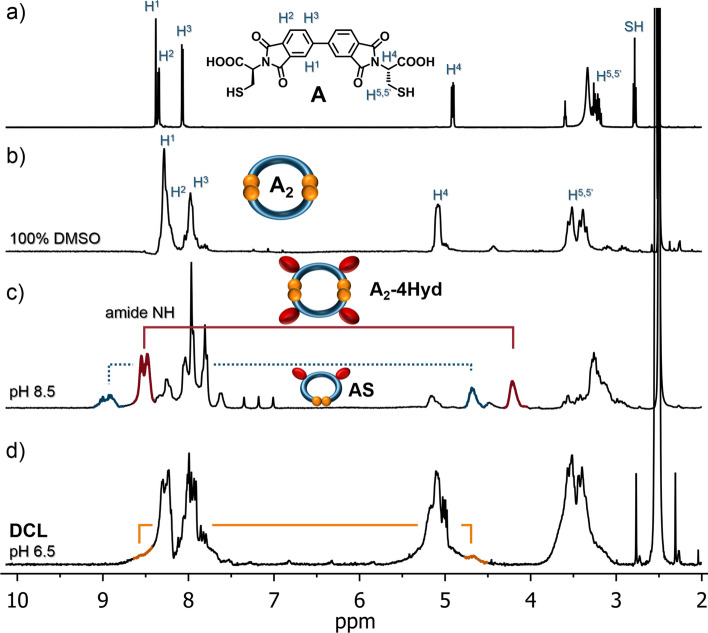
Comparison of ^1^H NMR spectra (300 MHz, 298 K, DMSO-d_6_) of (**a**) **A**, (**b**) **A**_**2**_, (**c**) **A**_**2**_**-4Hyd** (contaminated with **AS**) and (**d**) DCL mixture from Fig. 3f. Ties show coupled N–H signals with C-H signals (based on COSY, see Figs. S32–33).

By chemical intuition, one would expect a symmetrical product with a double *meta* or *para* configuration. Presumably, due to electronic and entropic factors, the system favors two-step hydrolysis path, which leads to an unsymmetrical product (for more details see Fig.S34). To the best of our knowledge, such directional hydrolysis has not been recorded previously for BPDA derivatives. After hydrolysis, the **A-2Hyd** should display extra structural flexibility. To check how this new feature of **A-2Hyd** influences the formation of disulfides, we set a new solution of **A** at pH 8.5 for oxidation with air exposure (Fig. 3a). The post-reaction mixture contained mostly one product (90%), which was identified by ^1^H NMR spectroscopy (Fig. 4c) and ESI–MS as the monoprotonated cation [M + H]^+^ remarkable cyclic *m-*, *p-*dimer with two disulfide bonds (**A**_**2**_**-4Hyd**) formed from two molecules of **A-2Hyd**. The second component **AS** (10%) is the monomeric **A-2Hyd** derivative cyclised by a single intramolecular disulfide bond.

To accelerate the oxidation, we used the known DMSO effect^15^. We started with **A** dissolved in pure DMSO (Fig. 4a) with expectation of rapid and full conversion^36,37^. After 3 days we observed via LC–MS the selective formation of only one product, which was identified as dimeric macrocycle **A**_**2**_ with two disulfide bonds (Fig. 3b). This compound was isolated and analyzed by NMR (Fig. 4b). The ^1^H spectrum of the dimer **A**_**2**_ shows the upfield shifts of aromatic signals characteristic of tight aromatic macrocycles, and downfield shifts of aliphatic C-H and CH_2_ signals. The absence of any amide signals indicates that no hydrolysis took place. A comparison of both **A**_**2**_ and **A**_**2**_**-Hyd** dimers shows that **A** has a structural tendency to form dimeric systems.

Here we also checked whether hydrolysis is possible after thiol oxidation. The isolated **A**_**2**_ was dissolved in water (5 mM, pH 8.5, r.t.) and monitored by HPLC. After 6 h the complete hydrolysis of **A**_**2**_ to **A**_**2**_**-4Hyd** occurred (Fig. 1, top). Placing the sample of **A**_**2**_**-4Hyd** in pure DMSO (5 mM, r.t.) did not lead to **A**_**2**_ diimide again, confirming the irreversible nature of the hydrolysis process under such conditions.

Based on the results so far, we decided to use a combination of pH control and DMSO in an attempt to expand to DCLs with larger macrocycles (Fig. 3c–f). To find the right conditions, we screened both concentration of DMSO and the pH in the range from 4.0 to 8.5 in 0.1 M AcONH_4_ buffer adjusted to the desired pH with AcOH and ammonia (see Figs. S28–30).

It became clear that pH had the greatest influence on the DCL product distribution. In this system, the most interesting results occur for pH 6.5 and 7.25, which fits with the usual optimal thiol oxidation conditions (Fig. 3e–f)^38^. pH 8.5 is usually applied to fully deprotonate the COOH groups and thus achieve water solubility although such a high pH is not optimal for the thiol oxidation. At lower pH values, the concentration of thiolate ion in the reaction mixture is lower, while its nucleophilicity (reactivity) is higher. Ultimately, we used a 5% DMSO solution in ammonium acetate buffer at pH 6.5, which provides much better conditions for the oxidation of thiols. We did not observe any significant changes in the library composition (pH 6.5) over the range 5–50% DMSO. However, the presence of DMSO can act as a switch that changes the resulting DCL composition substantially.

Our combined method with the appropriate pH and the DMSO as oxidation accelerator resulted in a multi-component library containing trimeric and tetrameric macrocycles. Based on LC–MS analysis (Fig. 3g), we found that in most cases (pH 5.0–7.25) a dynamic combinatorial library of six products was formed, which were identified as dimers, trimers and tetramers (as monoprotonated species [M + H]^+^). These are three pairs of dimeric (**A**_**2**_ and **A**_**2**_**-Hyd**), trimeric (**A**_**3**_ and **A**_**3**_**-Hyd**) and tetrameric (**A**_**4**_ and **A**_**4**_**-Hyd**) macrocycles, in which the first member is completely unhydrolyzed, while the second has one hydrolyzed imide group (based on MS). In the case of mono-hydrolyzed products, it should be noted that the ring-opening during the hydrolysis can take place either in *meta* or *para* position. However, these isomers are indistinguishable by LC–MS, so we were unable to determine exactly which isomers are observed in the generated DCLs.

We showed that the library composition can be controlled by pH. At slightly acidic pH (4.0), an unhydrolyzed dimer **A**_**2**_ dominates; a pH close to neutral (6.5–7.25) promotes the formation of the tetramer **A**_**4**_; while basic pH 8.5 causes the formation of a hydrolyzed dimer **A**_**2**_**-Hyd**. Due to similarity in the polarity of the library components, we were unable to isolate preparative amounts of separate products despite numerous attempts. Therefore we decided to analyze the intact DCL. The library was isolated by evaporation to dryness and analyzed by NMR (Fig. 4d). The ^1^H spectrum of the library contains significantly more signals (in comparison to **A** and **A**_**2**_), indicating a loss of symmetry of the trimeric and tetrameric products. In the COSY spectrum, tiny N–H signals coupled with the C-H signals (similar to **A**_**2**_**-Hyd**) are observed. This is additional proof for the presence of amides in the structures of some mono-hydrolyzed macrocycles in DCL.

**Figure 5:**
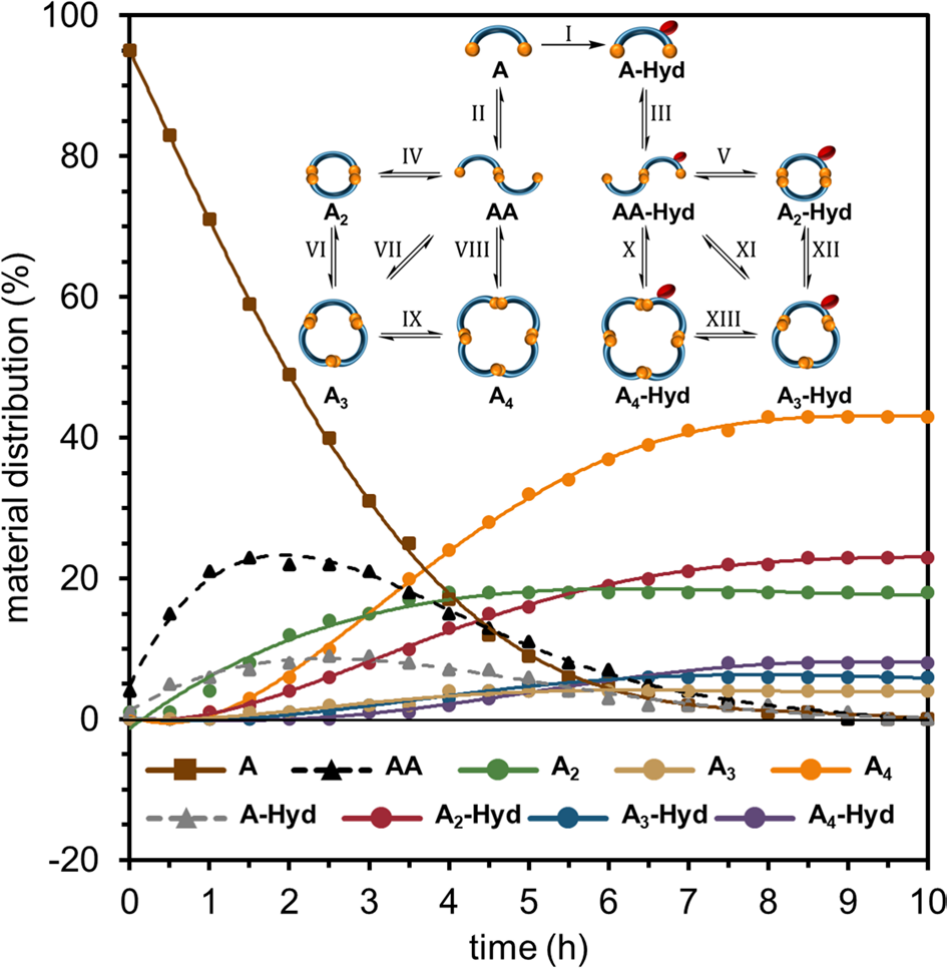
Kinetic plots of **A** DCL equilibration at pH 6.5 (substrate and products), and scheme of crucial reactions in the equilibrating mixture.

We were excited to get a deeper understanding of the system kinetics and mechanistic features. We employed HPLC to monitor the DCL equilibration over time (1 ml volume, pH 6.5, 5% DMSO, 5 mM of **A**). The DCL was analyzed every 30 min for 24 h at room temperature. The first injection (*t*_*0*_) has been done immediately after **A** was dissolved in the reaction buffer. The distribution curves of individual species during the equilibration were plotted, based on the integrations of the relative peak areas (RPA) from the collected chromatograms (Fig. 5).

It revealed that the system reaches equilibrium just after approx. 10 h and does not change any further up to 24 h. Several preliminary conclusions concerning the kinetics and mechanism of this complex process can be drawn. Firstly, substrate **A** (brown plot) irreversibly hydrolyzes to **A-Hyd** (reaction I, grey) and reach 10% abundance after approx. 2 h. Simultaneously, **A** dimerizes into a linear **AA** intermediate (II, black) with a single S–S bond and reaches maximum abundance of about 20% after approx. 1.5 h. Then **AA** undergoes intramolecular cyclization to macrocyclic **A**_**2**_ (IV, green). It seems that the synthesis of the larger macrocycles **A**_**3**_ (gold) and **A**_**4**_ (yellow) depends on the presence of **AA** and **A**_**2**_ (VI-IX) because they appear in the mixture only after 2 h. No traces of the expected **AAA** and **AAAA** linear intermediates were found in the obtained HPLC–MS data. However, intermediates **AAA** and **AAAA** seem to be an obvious step in chain elongation and ring closure processes. Therefore, it should be assumed that these individuals are formed in a mixture from the smaller molecules (**A** and **AA**) and are immediately consumed in the cyclization reaction to the macrocyclic products (**A**_**3**_ and **A**_**4**_). Similar results were observed for mono-hydrolyzed macrocycles, which seem to be strongly dependent from the **A-Hyd** concentration. The **A-Hyd** reacts with available **A** and forms **AA-Hyd** intermediate (III), which is rapidly intermolecularly cyclized into the major mono-hydrolyzed **A**_**2**_**-Hyd** macrocycle (V, red). The synthesis of the **A**_**3**_**-Hyd** (blue) and **A**_**4**_**-Hyd** (violet) may go through various pathways (X-XIII). It could also be observed that the DMSO accelerated oxidation and imides hydrolysis run in parallel with a slight vantage of first one during the first two hours. It confirms our main hypothesis that both reactions occur orthogonally during the entire process of DCL equilibration. The final ratio of the hydrolyzed to non-hydrolyzed products is approx. 3:6 (see Fig. 3f). The observed unusual imide hydrolysis in slightly acidic conditions may be powered by the presence of thiols. There are examples in the literature of the thiol-catalyzed hydrolysis of acyl groups^39,40^. We observed that the sample of unhydrolyzed **A**_**2**_ placed in pH 6.5 buffer has not changed over time (3 days). The addition of NaOH to pH 8.5 resulted in a rapid hydrolysis into the **A**_**2**_**-4Hyd** (as shown in Fig. 1). These results indicate that the hydrolysis in DCLs at pH 4–6.5 is dependent on the presence of unoxidized thiols. The latter significantly slow down the hydrolysis process or stop it completely when the oxidation is complete.

**Figure 6:**
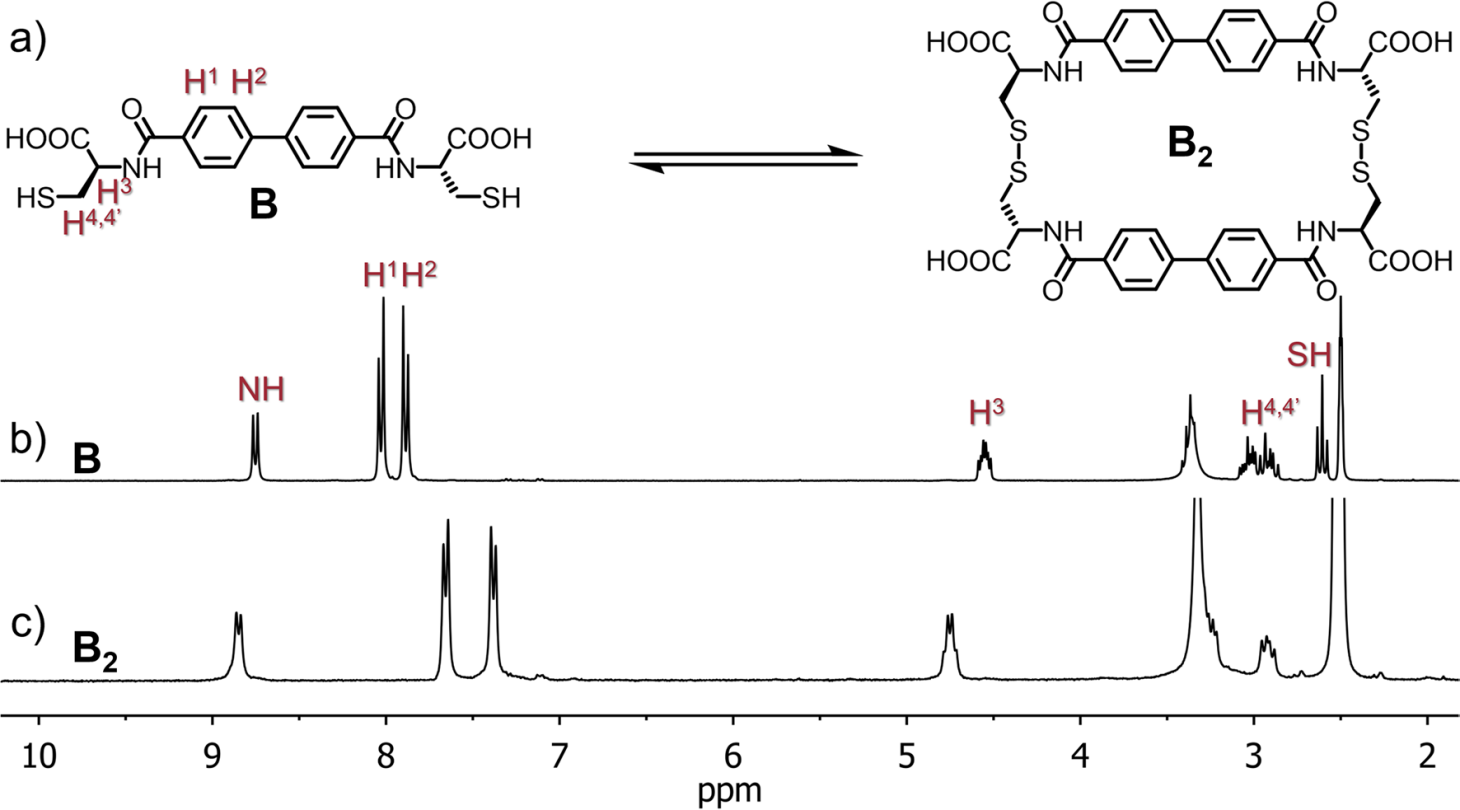
(**a**) synthesis scheme of the dimeric macrocycle **B**_**2**_ and comparison of ^1^H NMR spectra (300 MHz, 298 K, DMSO-d_6_) of (**b**) **B** and (**c**) **B**_**2**_**.**

Thus, we have shown that the use of a cysteine-modified phthalimide analogue gives unusual properties and allows the formation of multi-component DCLs. In order to demonstrate that the dynamics of the described system are strongly dependent on the hydrolysis of imides, and are not a property of the biphenyl structure, we performed a control experiment. We synthesized component **B** based on modified biphenyl-4,4′-dicarboxylic acid with two cysteine moieties (Fig. 6).

This compound is linear and has no imide groups but is otherwise analogous to component **A**. We repeated all the experiments that were described earlier with **A**. Under all pH and DMSO conditions only the cyclic dimer **B**_**2**_ was observed as the result of oxidation. Those experiments unequivocally confirmed the correlation between imide presence and component **A** activity.

## Conclusions

We have successfully demonstrated that the use of hydrolysable imides in dynamic disulfide chemistry can effectively act to enhance disulfide exchange factor for building multi-component DCLs. Additionally, we have shown that this effect could be externally modulated by pH and DMSO concentration. Until now, DMSO has been considered only as an oxidation accelerant, and we have shown that by changing the DMSO concentration, the library composition could be controlled. The presented methodology can be applied in the generation of new dynamic molecular transporters or receptors (e.g. cages or macrocycles) in which the water solubility could be modulated through a controlled process of imides hydrolysis. Finally, we delivered new insights into the kinetics and mechanistic properties of such type of complex disulfide system.

## Supplementary Information

Supplementary Information.

## References

[1] Zhang Y, Qi Y, Ulrich S, Barboiu M, Ramström O (2020). Dynamic covalent polymers for biomedical applications. Mater. Chem. Front..

[2] Phan N-M, Percástegui EG, Johnson DW (2020). Dynamic covalent chemistry as a facile route to unusual main-group thiolate assemblies and disulfide hoops and cages. ChemPlusChem.

[3] Ulrich S (2019). Growing prospects of dynamic covalent chemistry in delivery applications. Acc. Chem. Res..

[4] Corbett PT (2006). Dynamic combinatorial chemistry. Chem. Rev..

[5] Lehn JM (2007). From supramolecular chemistry towards constitutional dynamic chemistry and adaptive chemistry. Chem. Soc. Rev..

[6] Lehn J-M, Eliseev AV (2001). Dynamic combinatorial chemistry. Science.

[7] Nial JW, Craig RB, Collins JG, Sharon K, Janice RA-W (2007). DNA Intercalators in cancer therapy: Organic and inorganic drugs and their spectroscopic tools of analysis. Mini-Rev. Med. Chem..

[8] Bravin C, Guidetti A, Licini G, Zonta C (2019). Supramolecular cages as differential sensors for dicarboxylate anions: Guest length sensing using principal component analysis of ESI-MS and 1H-NMR raw data. Chem. Sci..

[9] Markiewicz G (2017). Selective C70 encapsulation by a robust octameric nanospheroid held together by 48 cooperative hydrogen bonds. Nat. Commun..

[10] Durot S, Taesch J, Heitz V (2014). Multiporphyrinic cages: Architectures and functions. Chem. Rev..

[11] Jędrzejewska H, Szumna A (2019). Peptide-based capsules with chirality-controlled functionalized interiors: Rational design and amplification from dynamic combinatorial libraries. Chem. Sci..

[12] Drożdż W (2018). Multivalent metallosupramolecular assemblies as effective DNA binding agents. Chem. Eur. J..

[13] Collins MS, Carnes ME, Nell BP, Zakharov LN, Johnson DW (2016). A facile route to old and new cyclophanes via self-assembly and capture. Nat. Commun..

[14] Phan N-M, Choy EPKL, Zakharov LN, Johnson DW (2019). Self-sorting in dynamic disulfide assembly: New biphenyl-bridged “nanohoops” and unsymmetrical cyclophanes. Chem. Commun..

[15] Atcher J, Alfonso I (2013). The effect of DMSO in the aqueous thiol–disulphide dynamic covalent chemistry of model pseudopeptides. RSC Adv..

[16] Ulatowski F, Sadowska-Kuzioła A, Jurczak J (2014). "Choose-a-size" approach in dynamic combinatorial chemistry: A single substrate dynamic combinatorial library of oligomacrocycles that adapts to the size and shape of carboxylates. J. Org. Chem..

[17] Sobczak S (2018). Dynamic covalent chemistry under high-pressure: A new route to disulfide metathesis. Chem. Eur. J..

[18] Fritze UF, von Delius M (2016). Dynamic disulfide metathesis induced by ultrasound. Chem. Comm..

[19] Belenguer AM, Michalchuk AAL, Lampronti GI, Sanders JKM (2019). Understanding the unexpected effect of frequency on the kinetics of a covalent reaction under ball-milling conditions. Beilstein J. Org. Chem..

[20] Ponnuswamy N, Cougnon FBL, Clough JM, Pantoş GD, Sanders JKM (2012). Discovery of an organic trefoil knot. Science.

[21] Ponnuswamy N, Cougnon FBL, Pantoş GD, Sanders JKM (2014). Homochiral and meso figure eight knots and a solomon link. J. Am. Chem. Soc..

[22] Dehkordi ME, Luxami V, Pantoş GD (2018). High-yielding synthesis of chiral donor-acceptor catenanes. J. Org. Chem..

[23] Prakasam T (2013). Simultaneous self-assembly of a [2]catenane, a trefoil knot, and a Solomon link from a simple pair of ligands. Angew. Chem. Int. Ed..

[24] Au-Yeung HY, Pantoş GD, Sanders JKM (2009). Amplifying different [2]catenanes in an aqueous donor−acceptor dynamic combinatorial library. J. Am. Chem. Soc..

[25] Lafuente M, Alfonso I, Solà J (2019). Structurally selective assembly of a specific macrobicycle from a dynamic library of pseudopeptidic disulfides. ChemSystemsChem.

[26] Fritze UF, Craig SL, von Delius M (2018). Disulfide-centered poly(methyl acrylates): Four different stimuli to cleave a polymer. J. Polym. Sci. A Polym. Chem..

[27] Stefankiewicz AR, Sanders JKM (2013). Diverse topologies in dynamic combinatorial libraries from tri- and mono-thiols in water: Sensitivity to weak supramolecular interactions. Chem. Commun..

[28] Drożdż W, Kołodziejski M, Markiewicz G, Jenczak A, Stefankiewicz RA (2015). Generation of a multicomponent library of disulfide donor-acceptor architectures using dynamic combinatorial chemistry. Int. J. Mol. Sci..

[29] Konopka M, Cecot P, Harrowfield JM, Stefankiewicz AR (2021). Structural self-sorting of pseudopeptide homo and heterodimeric disulfide cages in water: Mechanistic insights and cation sensing. J. Mater. Chem. C.

[30] Al Kobaisi M, Bhosale SV, Latham K, Raynor AM, Bhosale SV (2016). Functional naphthalene diimides: Synthesis, properties, and applications. Chem. Rev..

[31] Stefankiewicz AR, Tamanini E, Pantoş GD, Sanders JKM (2011). Proton-driven switching between receptors for C60 and C70. Angew. Chem. Int. Ed..

[32] Black SP, Sanders JKM, Stefankiewicz AR (2014). Disulfide exchange: Exposing supramolecular reactivity through dynamic covalent chemistry. Chem. Soc. Rev..

[33] Konopka M, Markiewicz G, Stefankiewicz AR (2018). Highly efficient one-step microwave-assisted synthesis of structurally diverse bis-substituted α-amino acid derived diimides. RSC Adv..

[34] Hasan SK, Abbas SA (1975). Alkaline hydrolysis products of n-substituted phthalimides. Can. J. Chem..

[35] Khan MN, Khan AA (1979). Kinetics and mechanism of base-catalysed hydrolysis of phthalimide. J. Chem. Soc. Perkin Trans..

[36] Drożdż W (2017). Generation of multicomponent molecular cages using simultaneous dynamic covalent reactions. Chem. Eur. J..

[37] Konopka M, Cecot P, Ulrich S, Stefankiewicz AR (2019). Tuning the solubility of self-assembled fluorescent aromatic cages using functionalized amino acid building blocks. Front. Chem..

[38] Nagy P (2012). Kinetics and mechanisms of thiol-disulfide exchange covering direct substitution and thiol oxidation-mediated pathways. Antioxid. Redox Signal..

[39] Hupe DJ, Jencks WP (1977). Nonlinear structure-reactivity correlations. Acyl transfer between sulfur and oxygen nucleophiles. J. Am. Chem. Soc..

[40] Llinás A (2000). Thiol-catalysed hydrolysis of benzylpenicillin. J. Chem. Soc Perkin Trans..

