# Supplementary material for: Expanding structural diversity in a library of disulfide macrocycles through in-situ imide hydrolysis
Source: Sci Rep. 2022 Jan 7;12:38. doi: 10.1038/s41598-021-03944-y (PMC8742088; doi:10.1038/s41598-021-03944-y)
Supplement: Supplementary file 1 — Supplementary Information. [file 41598_2021_3944_MOESM1_ESM.docx]

**Electronic Supplementary Information**

**Expanding Structural Diversity In A Library Of Disulfide Macrocycles Through *In-Situ* Imide Hydrolysis.**

Marcin Konopka ^a,b^ and Artur R. Stefankiewicz*^a,b^

a) Faculty of Chemistry, Adam Mickiewicz University, ul. Uniwersytetu Poznańskiego 8, 61-614 Poznań, Poland.

b) Centre for Advanced Technologies, Adam Mickiewicz University, ul. Uniwersytetu Poznańskiego 10, 61-614 Poznań, Poland.

Table of contents

Materials and methods 2

Synthesis of components 3

LC-MS Analysis 17

HPLC Data 27

References 34

# Materials and methods

All chemicals and solvents were purchased from commercial sources. NMR solvents were purchased from Deutero GmbH (Germany).

NMR spectra were acquired on Bruker Fourier 300 spectrometer equipped with 1H/13C 5 mm DUAL EasyProbe or Bruker Ascend 600 MHz equipped with 1H/13C 5 mm probe, and referenced on solvent residual peaks.

ESI-MS spectra were recorded on Bruker Impact HD Q-TOF spectrometer. HPLC measurements were performed on Hewlett Packard 1050 Series HPLC system coupled to a diode array detector.

LC-MS measurement were performed on UHPLC UltiMate 3000 Thermo Scientific/Dionex conjugated with Bruker Impact HD Q-TOF spectrometer. All LC separations were performed on the Synergi Phenomenex 4u POLAR-RP 80A, 75 x 4.60 mm, 4μm , with flow rate 0.5 ml/min in solvents gradient 10% MeCN in 90% H_2_O to 100% MeCN in 20 min. Solvents (water and acetonitrile) were acidified with 0.1% HCOOH. Chromatograms were acquired at 254 nm wave length.

HPLC measurements were performed on HP1050 series equipped with DAD UV-VIS detector. All HPLC separations were performed on the Synergi Phenomenex 4u POLAR-RP 80A, 75 x 4.60 mm, 4μm , with flow rate 1 ml/min in solvents gradient 10% MeCN in 90% H_2_O to 100% MeCN in 10 min. Solvents (water and acetonitrile) were acidified with 0.1% HCOOH. Chromatograms were acquired at 254 nm wave length.

A typical analytical DCL was prepared in a 1.0 ml scale by dissolving an equimolar mixture of thiol components (5 mM) in pH 6.5 buffer solution (5% DMSO in 0.1 M aqueous NH_4_OAc). The DCL was set in sealed HPLC 2 mL vial at room temperature until being analysed after 3 days. The pH of each library was checked before and after equilibration process to make sure it remained unchanged. The HPLC traces remained unchanged after 3 days indicating that thermodynamic equilibrium had been reached.

To ensure that the HPLC analyzes based on the relative peak area (RPA) are quantified, a series of UV-Vis concentration-dependent spectra was performed to compare the molar extinction coefficients between the substrate and the products.

All experiments containing DMSO were carried out in tightly sealed (otherwise unsealed) 2 ml vials in a 1.0 ml solution volume of 5 mM **A** or **B** solution, at r.t. The pH of each sample was checked before and after the experiment to make sure that it did not change over time. The samples were equilibrated for three days and the progress of the reaction was monitored by HPLC.

Synthesis of components

**Synthesis of A-STr ^[1]^**

The 3,3',4,4'-Biphenyltetracarboxylic dianhydride (1.47 g, 5.0 mmol), H-l-Cys-STr-OH (3.64 g, 10.0 mmol) and DMAP (2.44 g, 20.0 mmol) were dissolved in 50 mL of dry DMF in 100 mL round-bottom flask. The flask was equipped with an air condenser, placed in a microwave oven and heated at 140°C for 10 min under open vessel conditions. The post-reaction mixture was concentrated under vacuum and then poured into 200 mL of 1M HCl. The white precipitate was filtered off, washed with 500 mL of deionized water and dried under a high vacuum. Yield 4.23 g (86%) of **A-STr**.

**^1^H NMR** (600 MHz, DMSO-*d*_6_) *δ* 8.33 (dd, *J* = 24.6, 16.7 Hz, 4H), 8.04 (d, *J* = 7.7 Hz, 2H), 7.28–7.20 (m, 30H), 4.56 (dd, *J* = 11.4, 4.5 Hz, 2H), 3.09 (t, *J* = 12.9 Hz, 2H), 2.91 (dd, *J* = 13.1, 4.3 Hz, 2H). **^13^C NMR** (150 MHz, DMSO-*d*_6_) *δ* 168.86, 166.50, 143.97, 143.76, 129.02, 128.32, 128.10, 127.76, 127.50, 126.88, 126.22, 66.50, 55.77, 30.59. **ESI-MS**: *m*/*z* calculated for: [M+Na]^+^ 1007.2431, found: 1007.2240.

**Synthesis of A**

The **A-STr** (4.0 g, 4.0 mmol) was placed in a 100 mL round-bottom flask and dissolved under argon in a mixture of DCM 20 mL and trifluoroacetic acid (TFA) 5 mL. Then the Et_3_SiH (1.92 mL, 12.0 mmol) was added via syringe and the mixture was stirred at room temperature for 5 h. After, that the liquids were removed under vacuum and the resulting residue was suspended in 20 ml of Et_2_O and sonicated for a few minutes. The white precipitate was filtered off, washed with several portions of Et_2_O and dried under a high vacuum. Yield 1.90 g (93%) of **A**.

**^1^H NMR** (300 MHz, DMSO-*d*_6_) δ 13.49 (s, 1H), 8.54 – 8.25 (m, 2H), 8.07 (d, *J* = 7.8 Hz, 1H), 4.92 (dd, *J* = 10.2, 5.3 Hz, 1H), 3.33 – 3.13 (m, 2H), 2.79 (t, *J* = 8.7 Hz, 1H). **^13^C NMR** (75 MHz, DMSO-*d*_6_) δ 169.21, 166.95, 144.73, 134.10, 132.19, 130.93, 124.23, 122.69, 54.82, 22.99. **ESI-MS**: *m*/*z* calculated for: [M+H]^+^ 501.0420, found: 501.0482, calculated for: [M+Na]^+^ 523.0240, found: 523.0298.


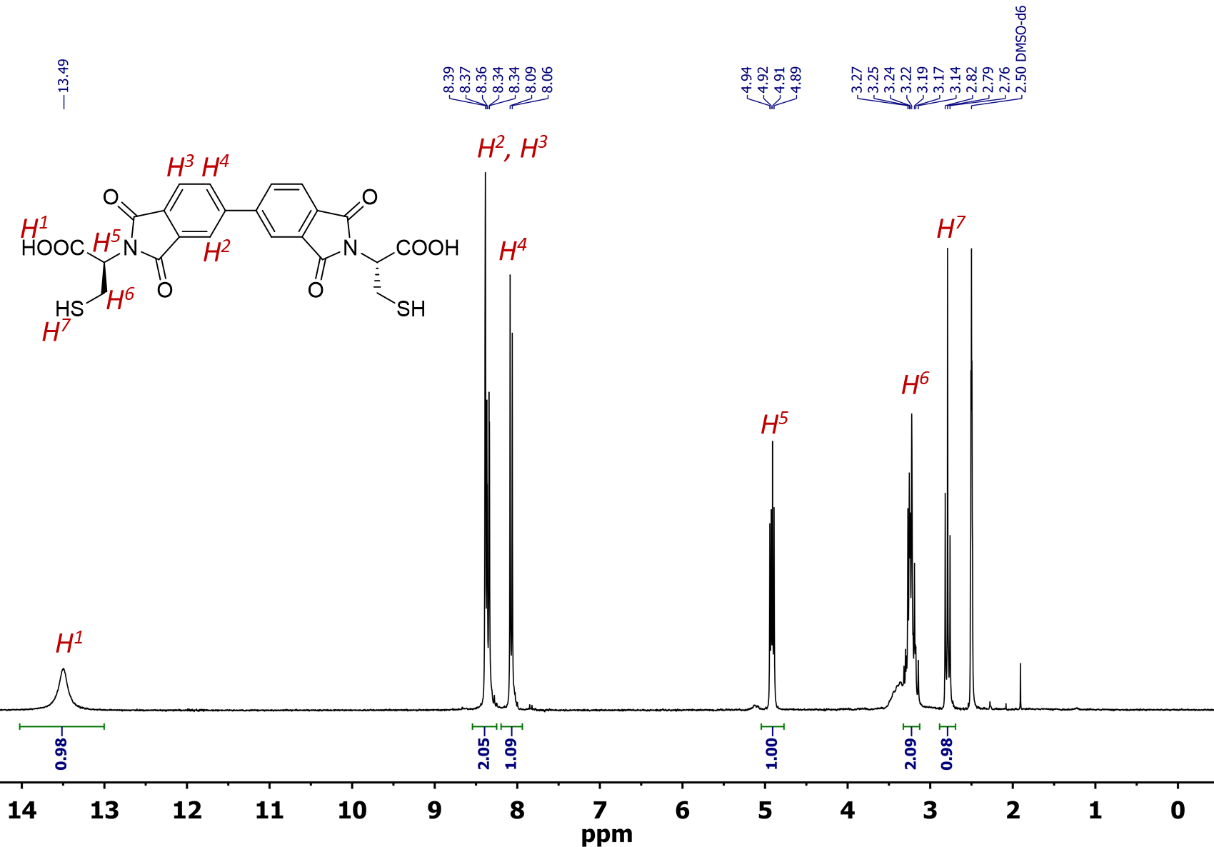


**Figure S1:** ^1^H NMR spectrum of component **A** in DMSO-*d_6_* at 298 K (300 MHz).


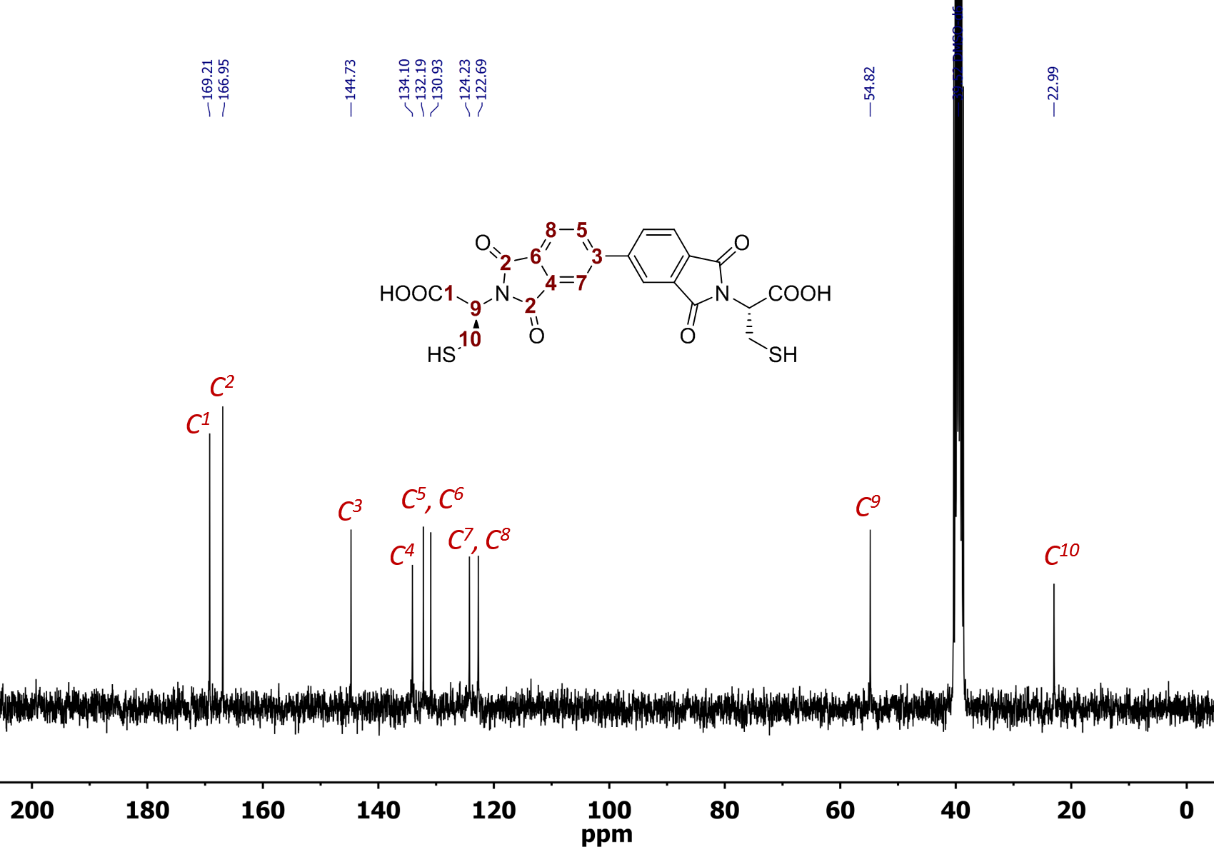


**Figure S2:** ^13^C NMR spectrum of component **A** in DMSO-*d_6_* at 298 K (75 MHz).


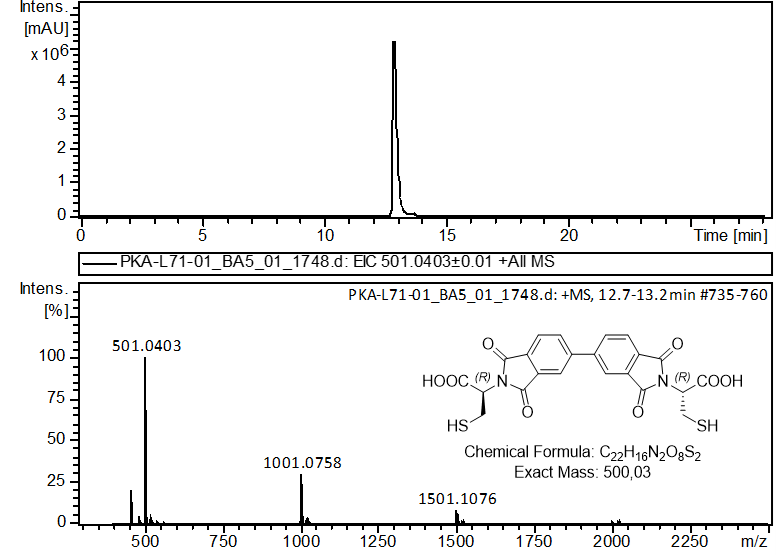


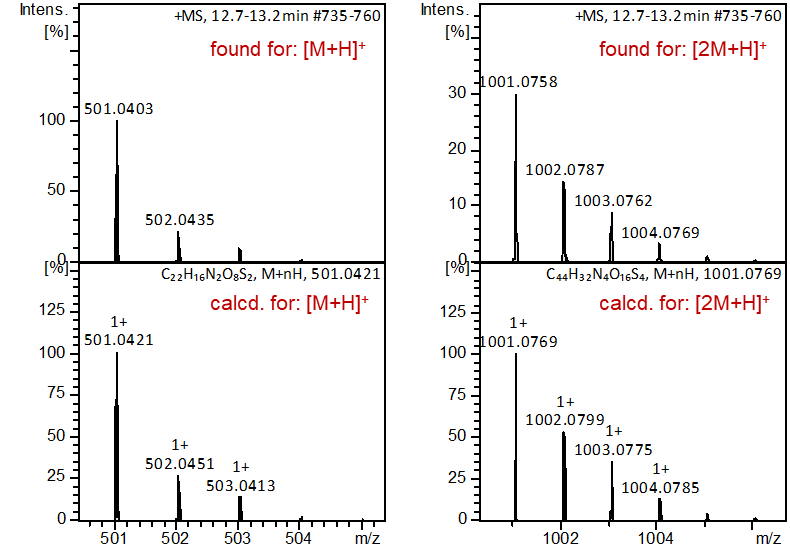


**Figure S3:** LC-MS analysis of **A**. Extracted ion chromatogram, +ESI-MS spectrum, simulated/found spectra comparison.

**Synthesis of B-EA^[2]^**

The Biphenyl-4,4'-dicarboxylic acid (2.42 g, 10 mmol) and *N*-hydroxysuccinimide (3.45 g, 30 mmol) were dissolved in dry DMF (100 mL) in a 250 mL round-bottom flask. After 15 min. of stirring the EDC·HCl (5.77 g, 30 mmol) was added stepwise and then the stirring was continued for 24 h at r.t. Then the solvent was removed under vacuum. To the oily residue, the 200 mL of 1M HCl was added and stirred for 10 min. The white precipitate was filtered off, washed with an additional 500 mL of deionized water and dried under a high vacuum. Yield 3.54 g (81%) of **B-EA**.

**^1^H NMR** (300 MHz, DMSO-*d*_6_) δ 8.24 (d, *J* = 8.6 Hz, 4H), 8.08 (d, *J* = 8.6 Hz, 4H), 2.92 (s, 8H). **^13^C NMR** (75 MHz, DMSO-*d*_6_) δ 170.32, 161.52, 144.80, 130.82, 128.30, 124.45, 25.59. **ESI-MS**: *m*/*z* calculated for: [M+Na]^+^ 459.0799, found: 459.0805.

**Synthesis of B-STr^[2]^**

To the solution of **B-EA** (3.5 g, 8.0 mmol) in dry DMF (150 mL), H-l-Cys-STr-OH (8.75 g, 24.0 mmol) and Et_3_N (3.35 mL, 40 mmol) were added. The mixture was stirred at room temperature for 24 h under a argon atmosphere. Then the solvent was removed under vacuum. To the oily residue, the 200 mL of 1M HCl was added and then stirred for 10 min. The white precipitate was filtered off, washed with an additional 500 mL of deionized water and dried under a high vacuum. Yield 6.50 g (87%) of **B-STr**.

**^1^H NMR** (300 MHz, DMSO-*d*_6_) δ 8.82 (d, *J* = 7.9 Hz, 2H), 8.00 (d, *J* = 8.4 Hz, 4H), 7.88 (d, *J* = 8.4 Hz, 4H), 7.40 – 7.21 (m, 30H), 4.33 (dq, *J* = 8.0, 4.7 Hz, 2H), 3.01 (q, *J* = 7.3 Hz, 2H), 2.84 – 2.66 (m, 2H). **^13^C NMR** (75 MHz, DMSO-*d*_6_) δ 179.69, 176.68, 144.35, 144.34, 129.15, 129.13, 129.11, 128.15, 128.08, 126.80, 120.60, 76.95, 66.14, 33.07. **ESI-MS**: *m*/*z* calculated for: [M+Na]^+^ 955.2846, found: 955.2796.

**Synthesis of B**

The **B-STr** (1.86 g, 2.0 mmol) was placed in a 100 mL round-bottom flask and dissolved under argon in a mixture of DCM 10 mL and trifluoroacetic acid (TFA) 2.5 mL. Then the Et_3_SiH (0.48 mL, 3.0 mmol) was added via syringe and the mixture was stirred at room temperature for 5 h. After, that the liquids were removed under vacuum and the resulting residue was suspended in 20 ml of Et_2_O and sonicated for a few minutes. The white precipitate was filtered off, washed with several portions of Et_2_O and dried under a high vacuum. Yield 0.86 g (96%) of **B**.

**^1^H NMR** (300 MHz, DMSO-*d*_6_) δ 8.75 (d, *J* = 7.8 Hz, 1H), 8.03 (d, *J* = 8.4 Hz, 2H), 7.89 (d, *J* = 8.5 Hz, 2H), 4.62 – 4.48 (m, 1H), 3.06 (dd, *J* = 15.3, 8.3 Hz, 1H), 2.97 – 2.86 (m, 1H), 2.61 (t, *J* = 8.3 Hz, 1H). **^13^C NMR** (75 MHz, DMSO-*d*_6_) δ 171.86, 166.11, 141.92, 133.19, 128.23, 126.79, 55.60, 25.24. **ESI-MS**: *m*/*z* calculated for: [M+H]^+^ 449.0836, found: 449.0886, calculated for: [M+Na]^+^ 471.0655, found: 471.0668.


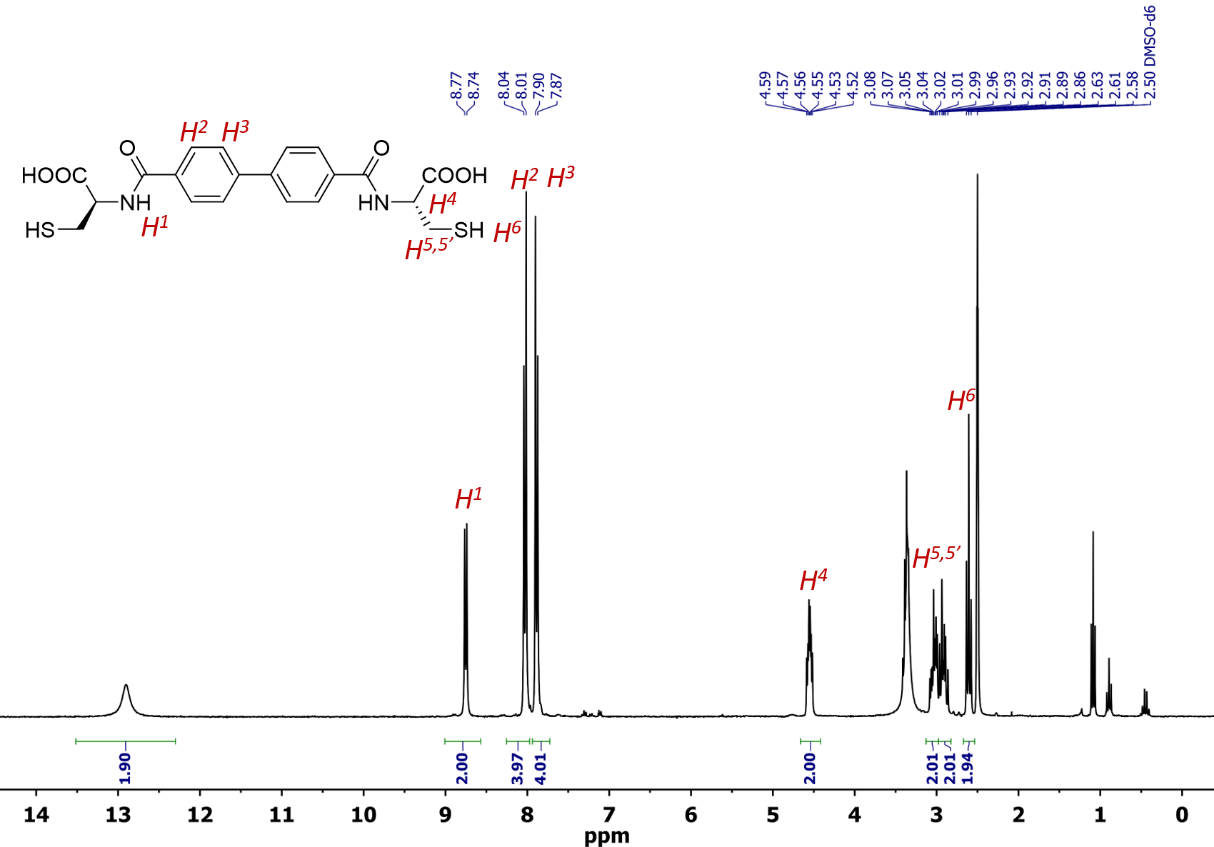


**Figure S4:** ^1^H NMR spectrum of component **1** in DMSO-*d_6_* at 298 K (300 MHz).


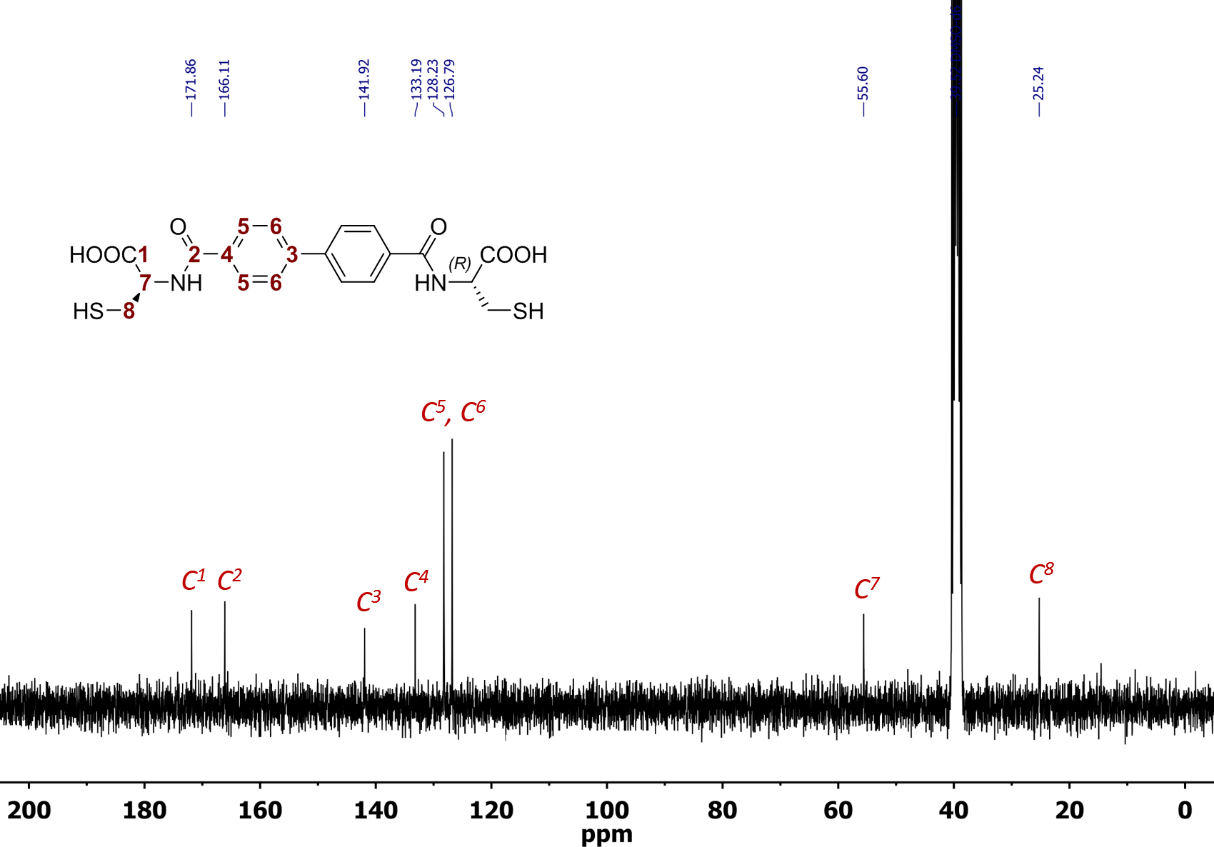


**Figure S5:** ^13^C NMR spectrum of component **A** in DMSO-*d_6_* at 298 K (75 MHz).


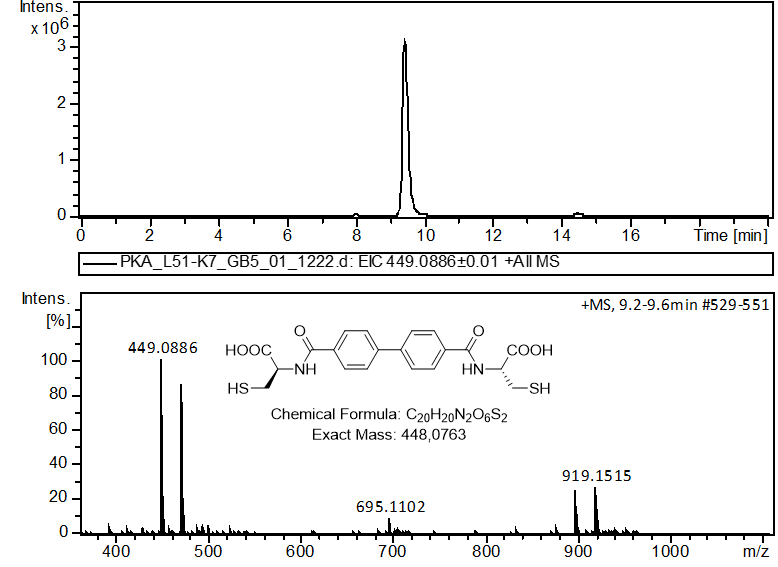


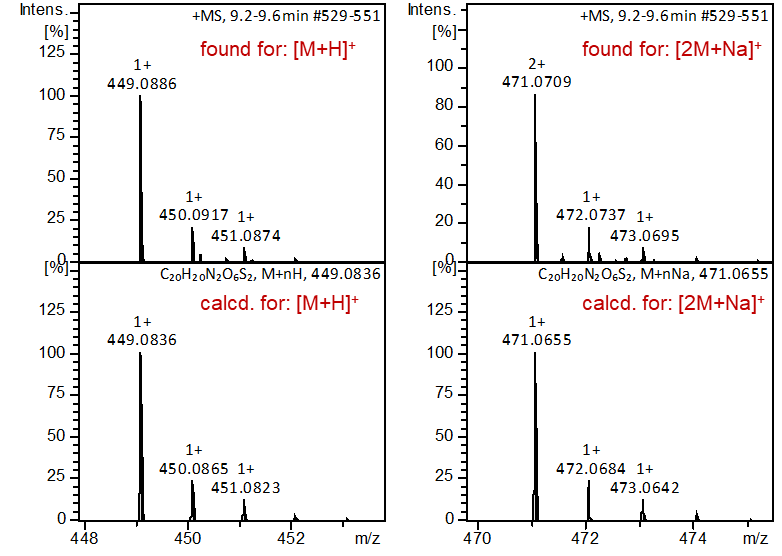


**Figure S6:** LC-MS analysis of component **B**. Extracted ion chromatogram, +ESI-MS spectrum, simulated/found spectra comparison.

**Synthesis of A-2Hyd**

The 100 mg of **A** was dissolved in 1mL of 1M NaOH in the vial and stirred. After 20 minutes, the mixture was acidified with 1 mL of concentrated aqueous HCl. The precipitated white solid was filtered off, washed with water and Et_2_O, and then dried under high vacuum. Yield 98 g (99%) of **A-2Hyd**.

**^1^H NMR** (300 MHz, DMSO-*d*_6_) δ 12.83 (s, 2H), 8.84 (dd, *J* = 7.9, 3.1 Hz, 1H), 8.74 (d, *J* = 7.9 Hz, 1H), 8.08 (dd, *J* = 8.3, 1.7 Hz, 1H), 7.99 (d, *J* = 8.0 Hz, 1H), 7.90 (s, 2H), 7.76 (d, *J* = 4.9 Hz, 1H), 7.61 (dd, *J* = 7.9, 4.5 Hz, 1H), 4.60 (qd, *J* = 7.6, 7.0, 3.0 Hz, 2H), 2.92 (tdd, *J* = 15.7, 13.8, 8.1 Hz, 4H), 2.61 – 2.51 (t, 2H). **^13^C NMR** (75 MHz, DMSO-*d*_6_) δ 172.15, 171.84, 168.53, 168.12, 141.43, 141.38, 132.36, 132.28, 130.54, 129.99, 129.86, 129.47, 129.35, 128.37, 126.85, 126.71, 55.59, 39.89, 25.83. **ESI-MS**: *m*/*z* calculated for: [M+H]^+^ 537.0632, found: 537.0624.


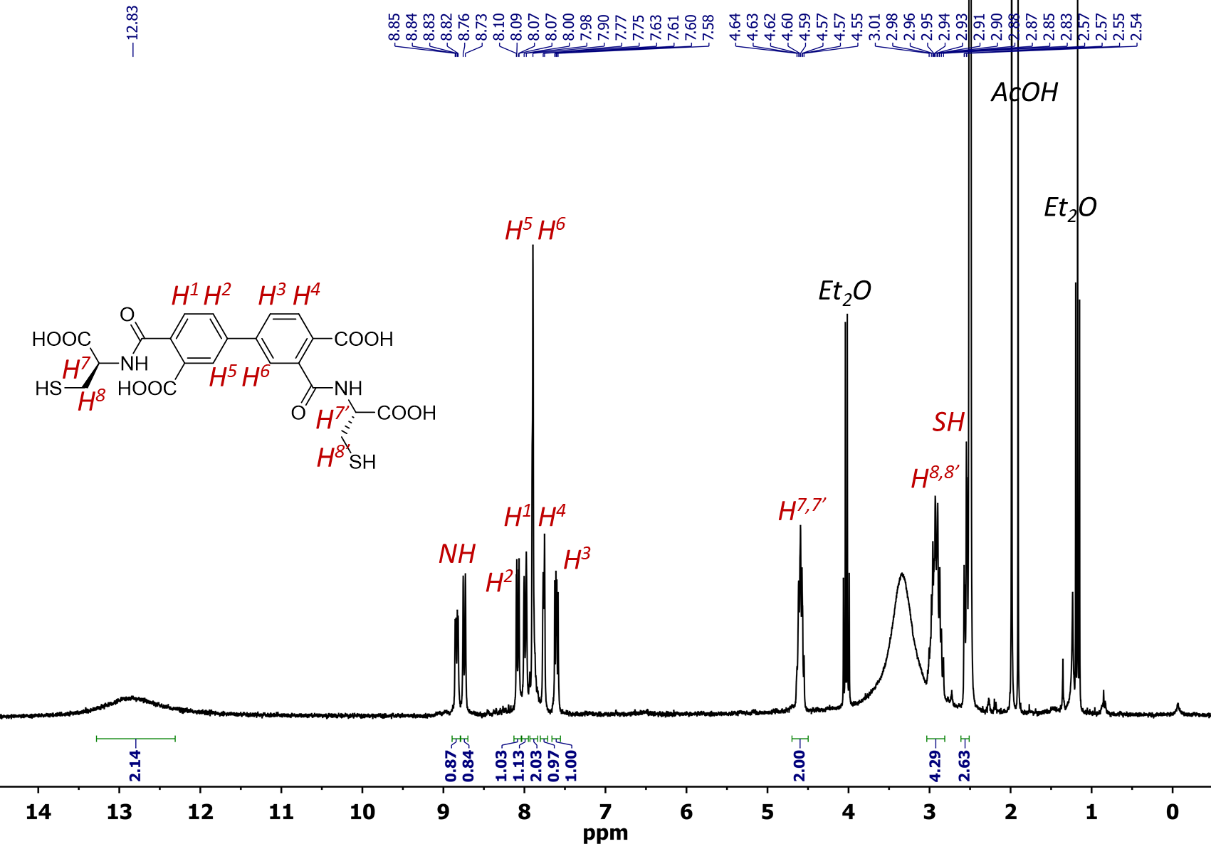


**Figure S7:** ^1^H NMR spectrum of **A-2Hyd** in DMSO-*d_6_* at 298 K (300 MHz).


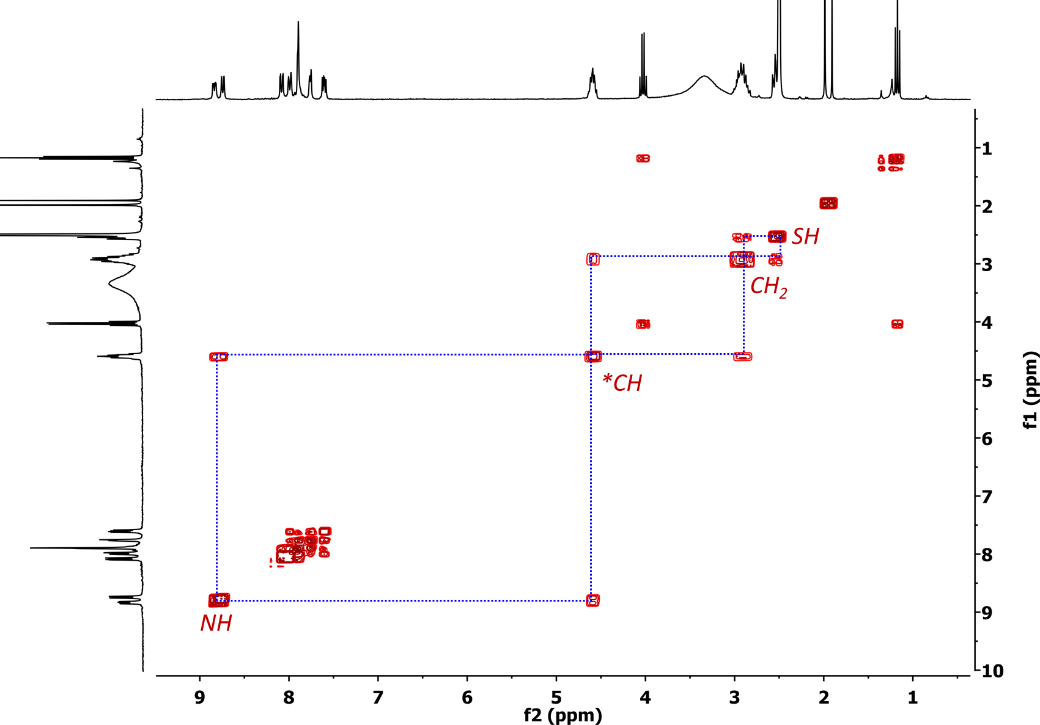


**Figure S8:** COSY NMR spectrum of **A-2Hyd** in DMSO-*d_6_* at 298 K (300 MHz).


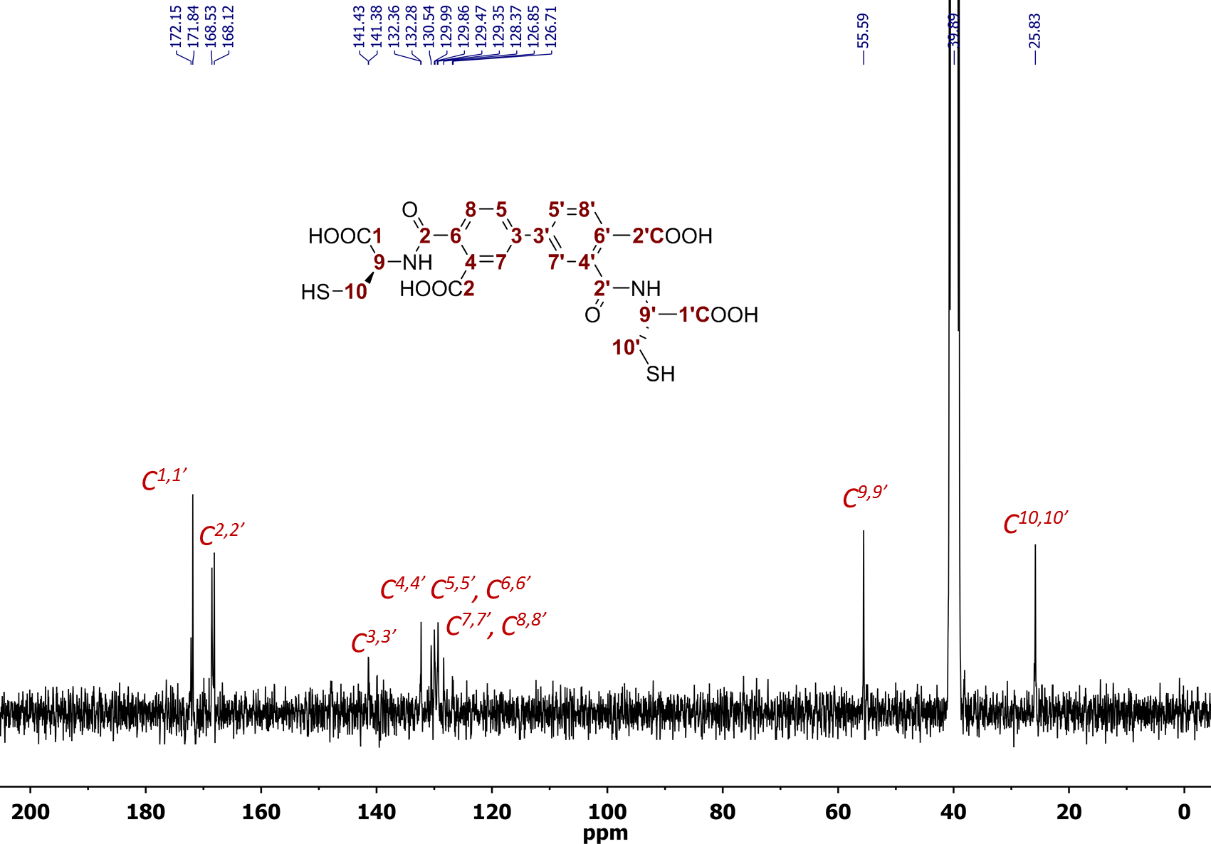


**Figure S9:** ^13^C NMR spectrum of component **A-2Hyd** in DMSO-*d_6_* at 298 K (75 MHz).


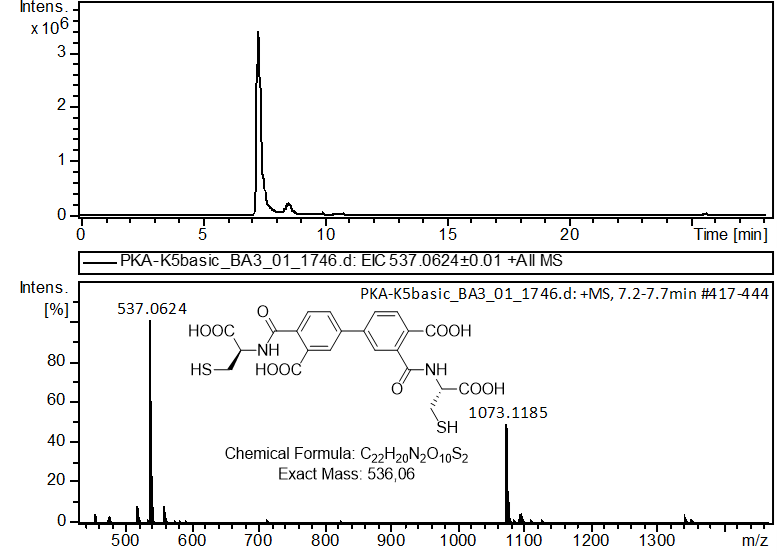


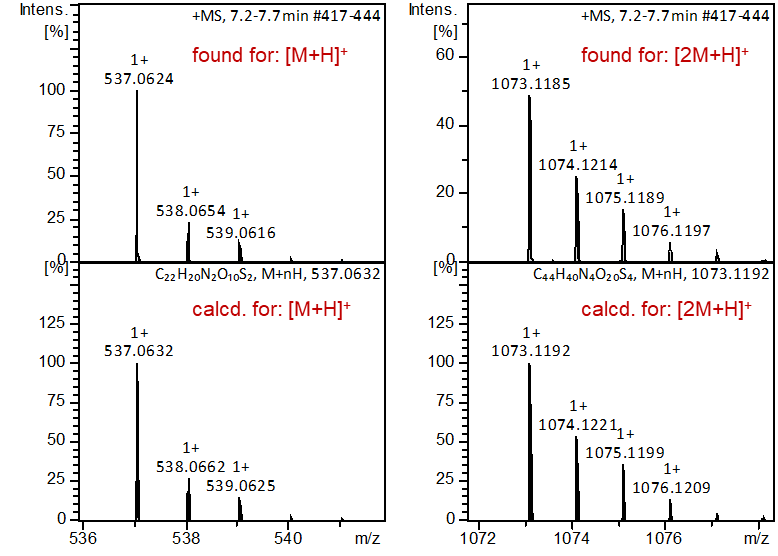


**Figure S10:** LC-MS analysis of **A-2Hyd**. Extracted ion chromatogram, +ESI-MS spectrum, simulated/found spectra comparison.

**Synthesis of A_2_**

The 50 mg of **A** (0.1 mmol) was dissolved in 1mL of DMSO and stirred at r.t. for 3 days. After that 20 mL of cold Et_2_O was added with vigorous stirring to precipitate the product. The white precipitate was centrifuged several times, each time washed with 10 mL of Et_2_O. Then it was dried under a high vacuum. Yield 35 g (70%) of **A_2_**.

**^1^H NMR** (300 MHz, DMSO-*d*_6_) δ 8.28 (s, 9H), 8.06 – 7.94 (m, 5H), 5.10 (q, *J* = 8.7, 5.4 Hz, 4H), 3.54 (d, *J* = 14.0 Hz, 4H), 3.38 (t, *J* = 12.1 Hz, 4H). **^13^C NMR** (75 MHz, DMSO-*d_6_*) δ 172.13, 169.31, 166.78, 144.76, 134.29, 132.00, 130.75, 124.35, 122.87, 51.26, 21.21. **ESI-MS**: *m*/*z* calculated for: [M+H]^+^ 997.0456, found: 997.0485.


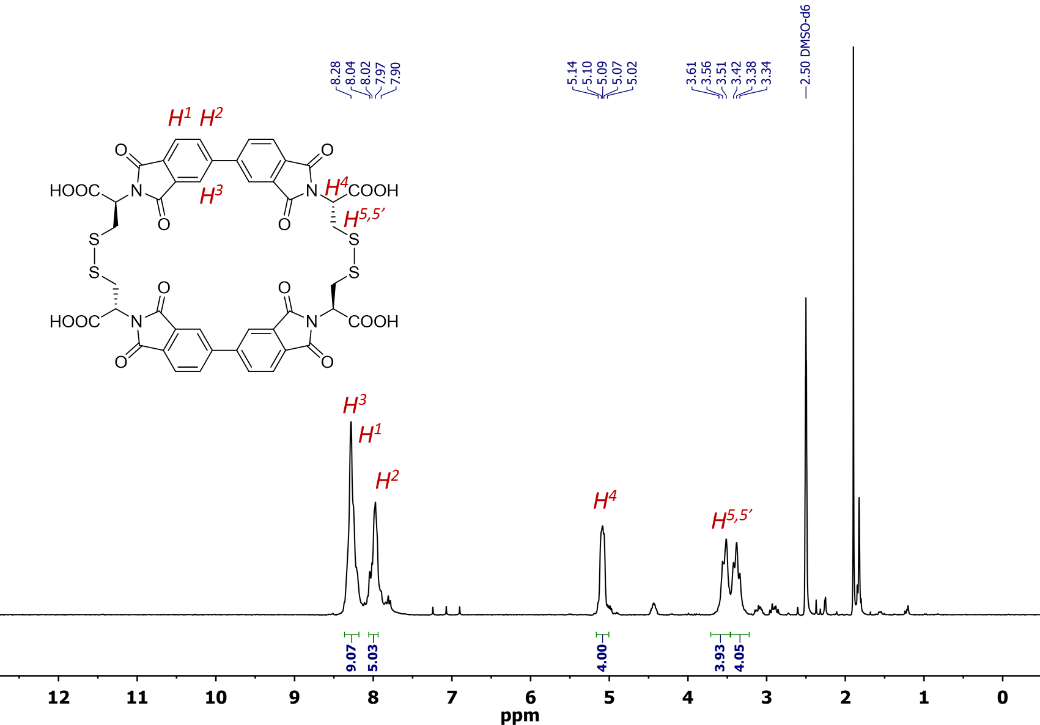


**Figure S11:** ^1^H NMR spectrum of **A_2_** in DMSO-*d_6_* at 298 K (300 MHz).


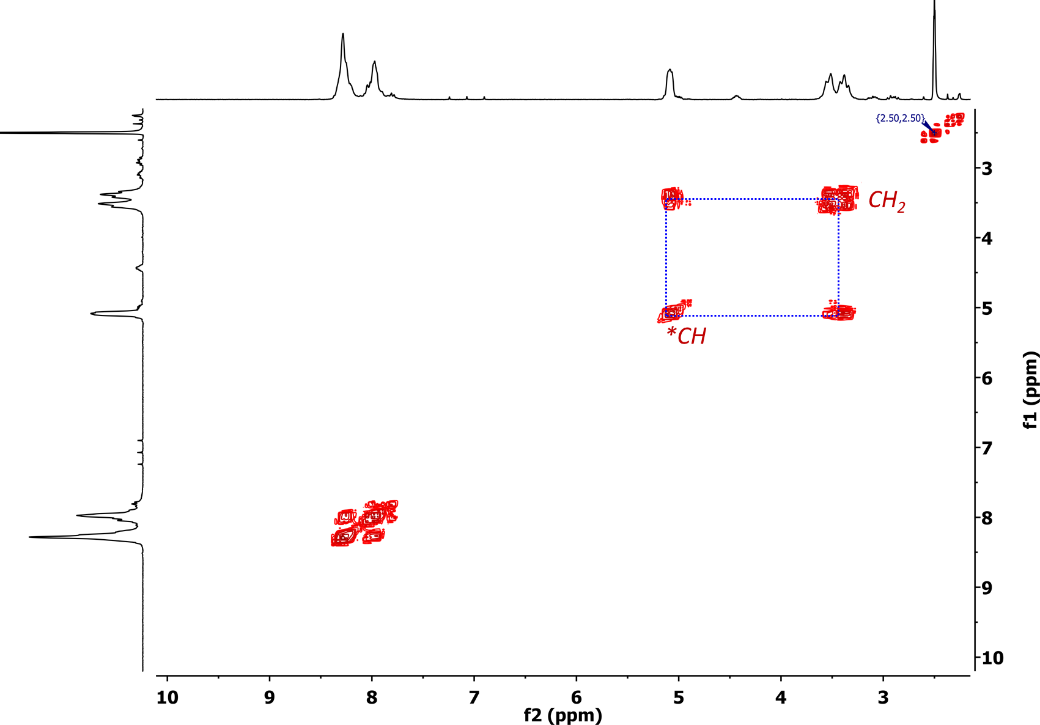


**Figure S12:** COSY NMR spectrum of **A_2_** in DMSO-*d_6_* at 298 K (300 MHz).


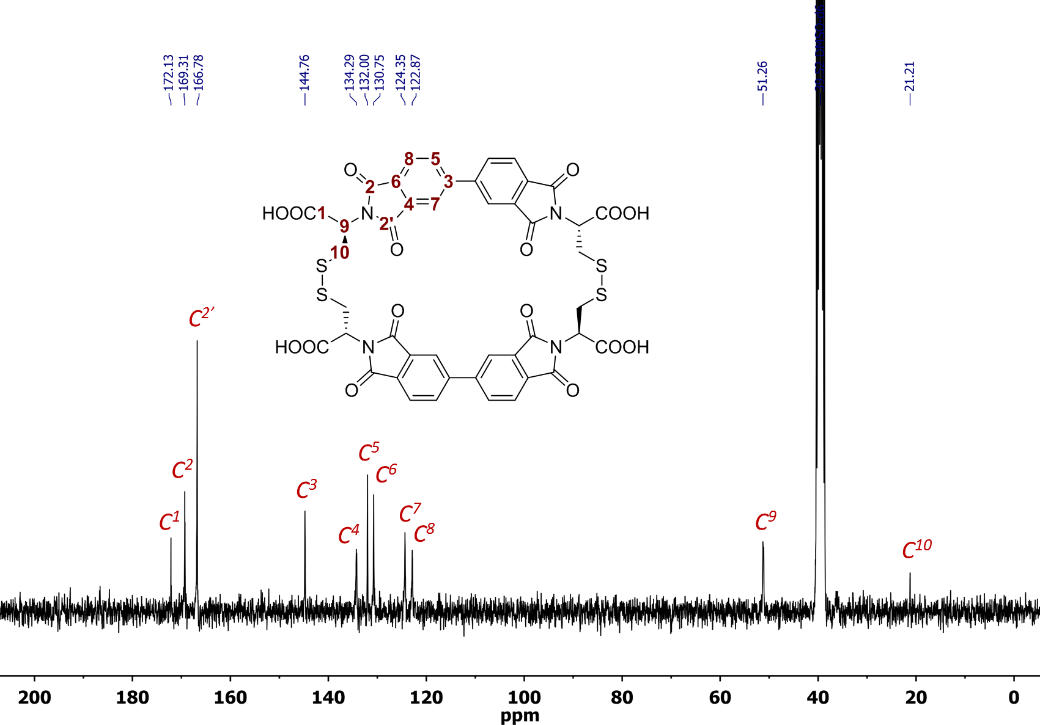


**Figure S13:** ^13^C NMR spectrum of component **A_2_** in DMSO-*d_6_* at 298 K (75 MHz).

**Synthesis of B_2_**

The 50 mg of B was dissolved in 2mL of a mixture of 0.1M AcONH_4_ buffer and 5% DMSO at pH 6.5 and stirred at rt. overnight. The mixture was then acidified with 1 mL of 1M HCl. The precipitated white solid was filtered off, washed with water and Et_2_O, and then dried under a high vacuum. Yield 46 g (92%) of **B_2_**.

**^1^H NMR** (300 MHz, DMSO-*d*_6_) δ 12.89 (s, 4H), 8.85 (d, *J* = 7.4 Hz, 4H), 7.66 (d, *J* = 7.9 Hz, 8H), 7.38 (d, *J* = 8.1 Hz, 8H), 4.75 (q, *J* = 7.4 Hz, 4H), 3.29 – 3.18 (m, 4H), 2.92 (dd, *J* = 13.5, 8.1 Hz, 4H). **^13^C NMR** (75 MHz, DMSO-*d_6_*) δ 172.20, 166.12, 141.96, 133.10, 128.23, 126.86, 51.97, 15.33. **ESI-MS**: *m*/*z* calculated for: [M+H]^+^ 893.1285, found: 893.1251.


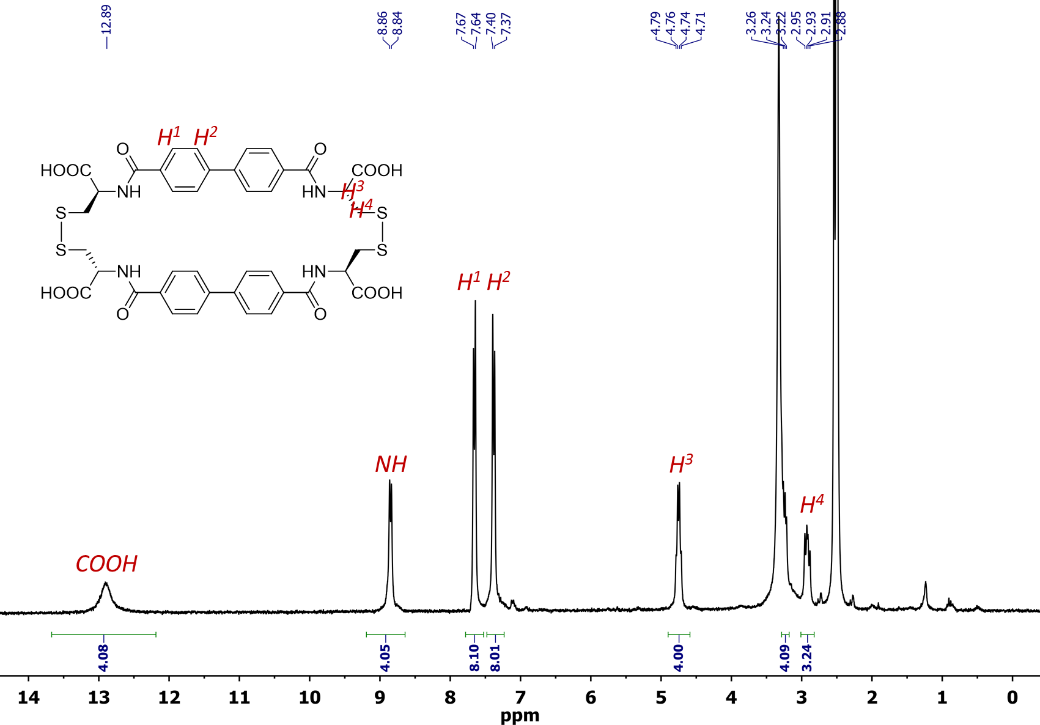


**Figure S14:** ^1^H NMR spectrum of **B_2_** in DMSO-*d_6_* at 298 K (300 MHz).


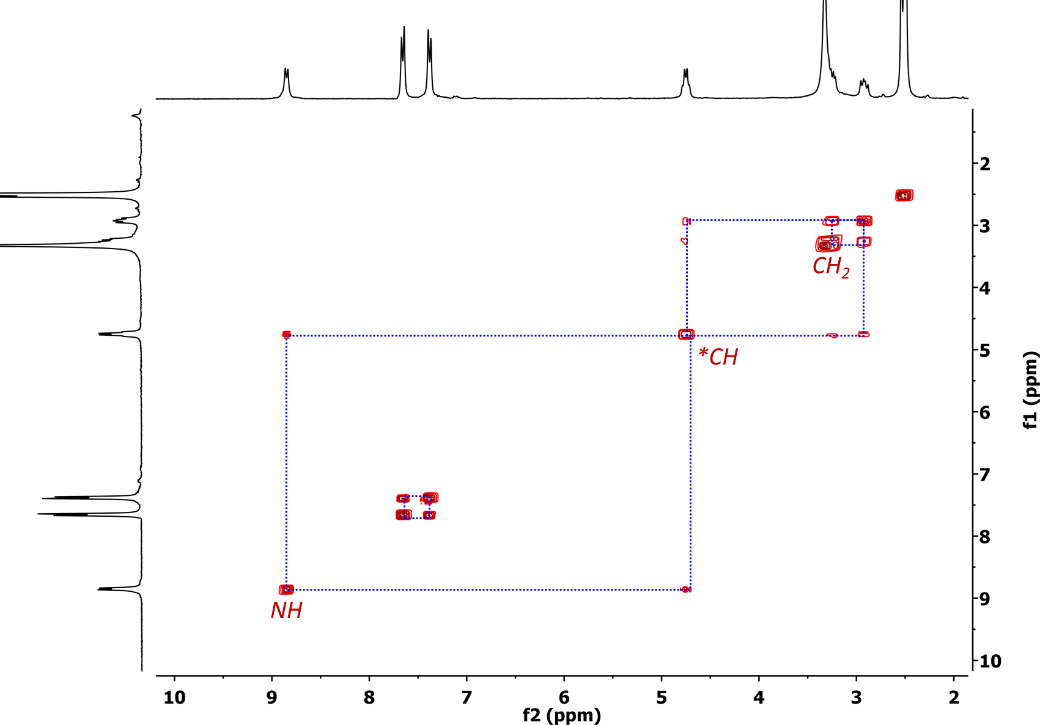


**Figure S15:** COSY NMR spectrum of **B_2_** in DMSO-*d_6_* at 298 K (300 MHz).


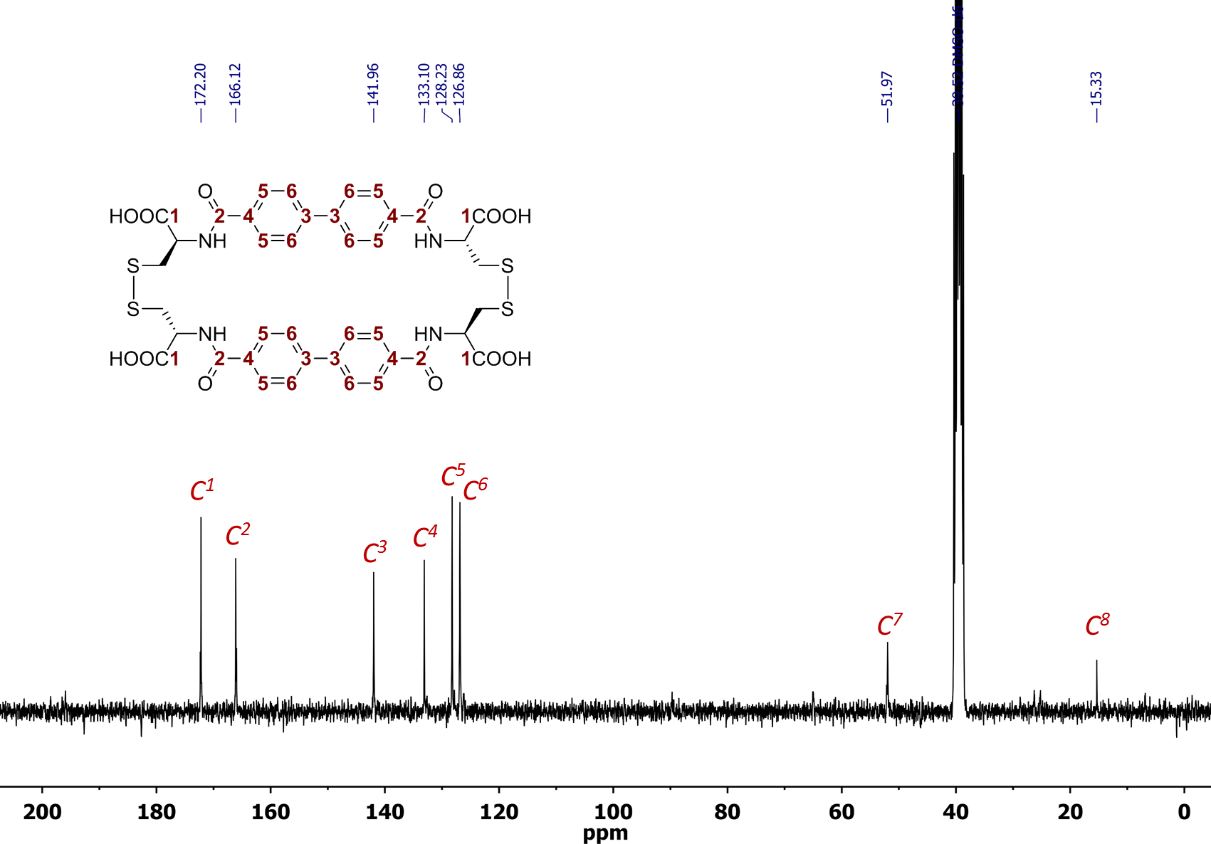


**Figure S16:** ^13^C NMR spectrum of **B_2_** in DMSO-*d_6_* at 298 K (75 MHz).


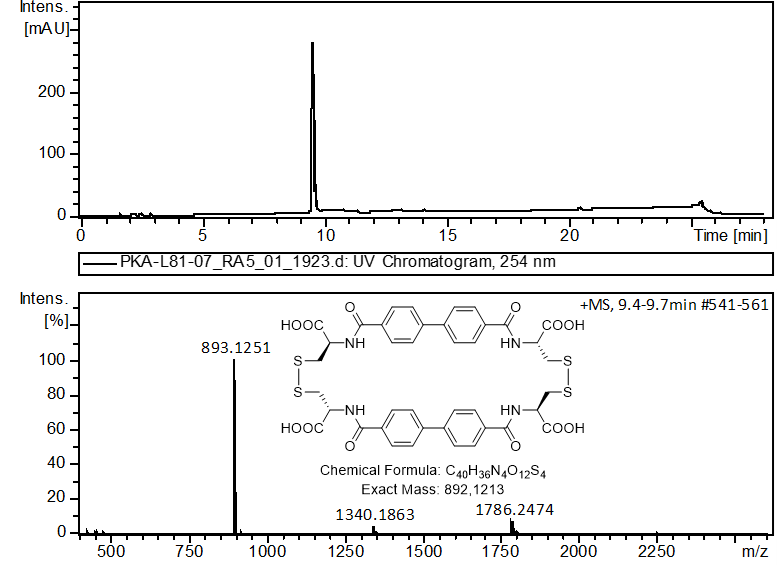


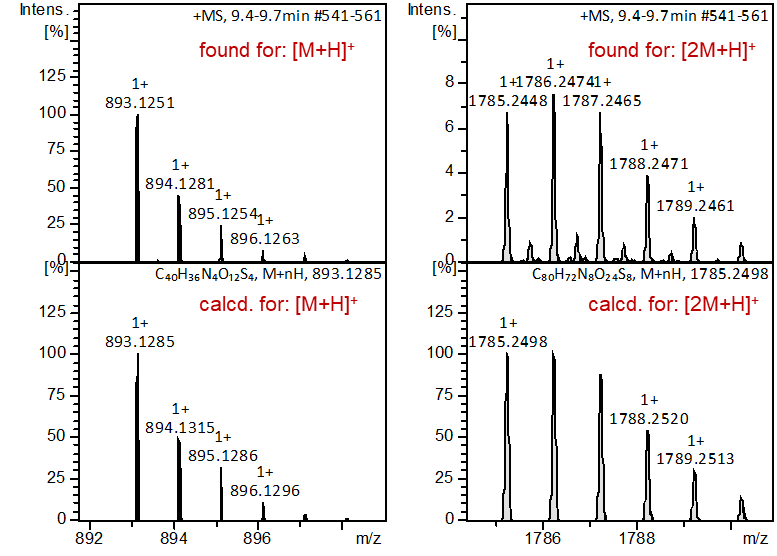


**Figure S17:** LC-MS analysis of **B_2_**. Extracted ion chromatogram, +ESI-MS spectrum, simulated/found spectra comparison.

# LC-MS Analysis

**A_2_**


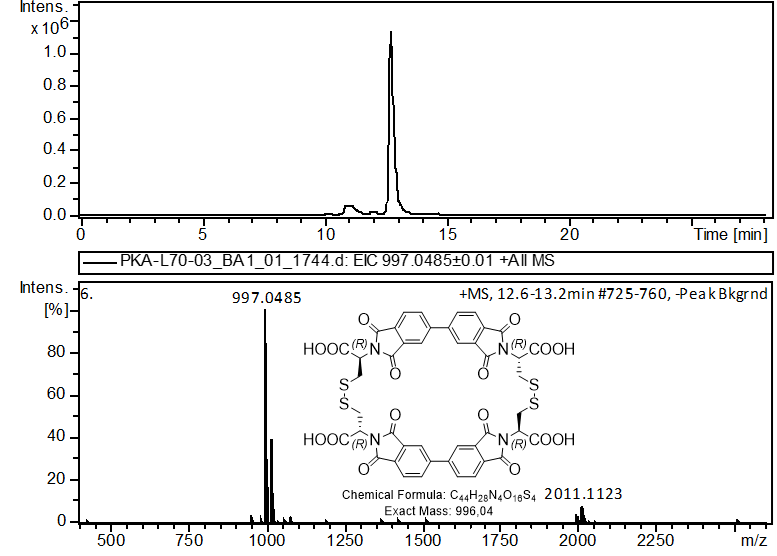


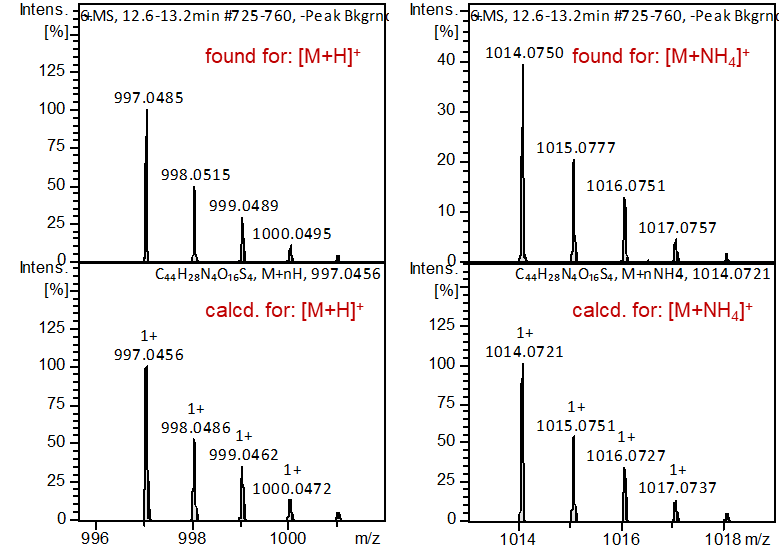


**Figure S18:** LC-MS analysis of **A_2_**. Extracted ion chromatogram, +ESI-MS spectrum, simulated/found spectra comparison.

**A_2_-Hyd**


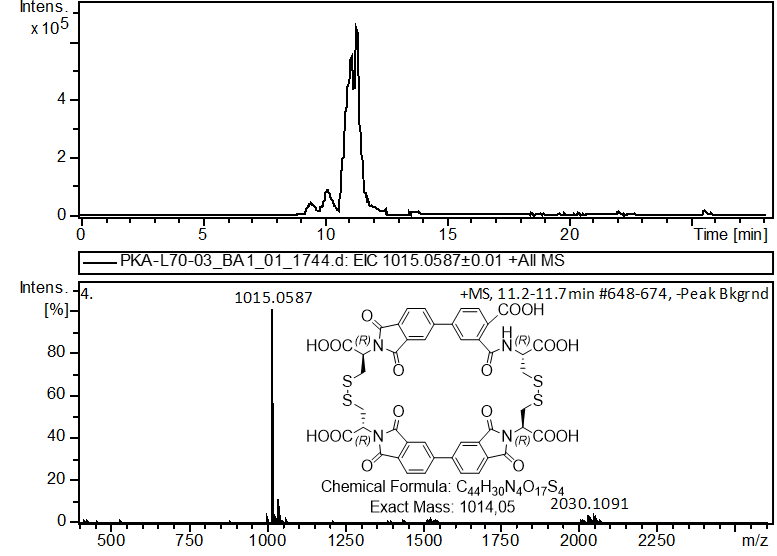


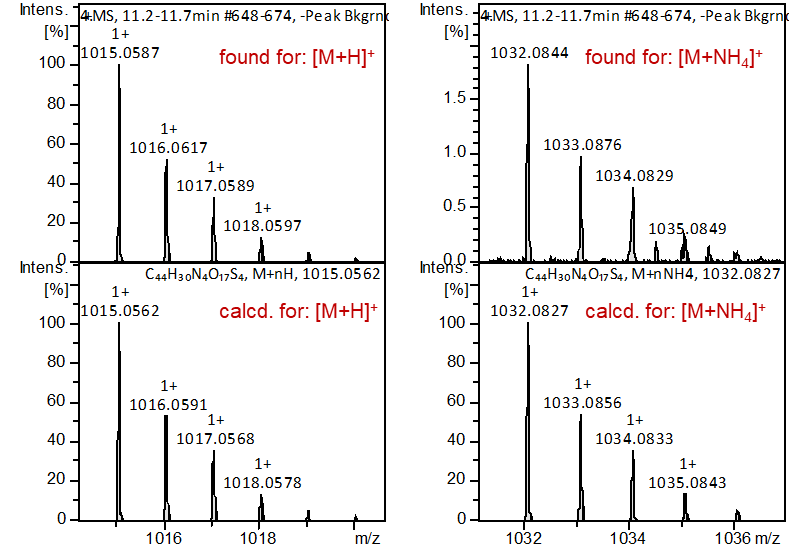


**Figure S19:** LC-MS analysis of **A_2_-Hyd**. Extracted ion chromatogram, +ESI-MS spectrum, simulated/found spectra comparison.

**A_2_-4Hyd**


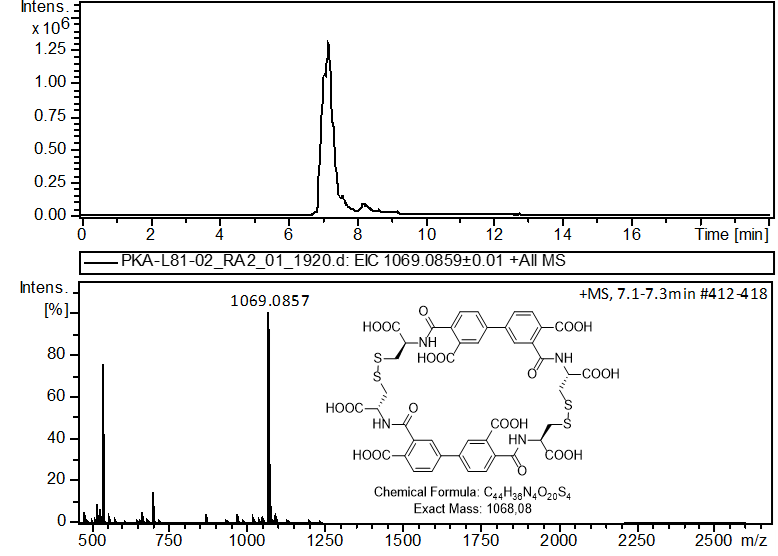


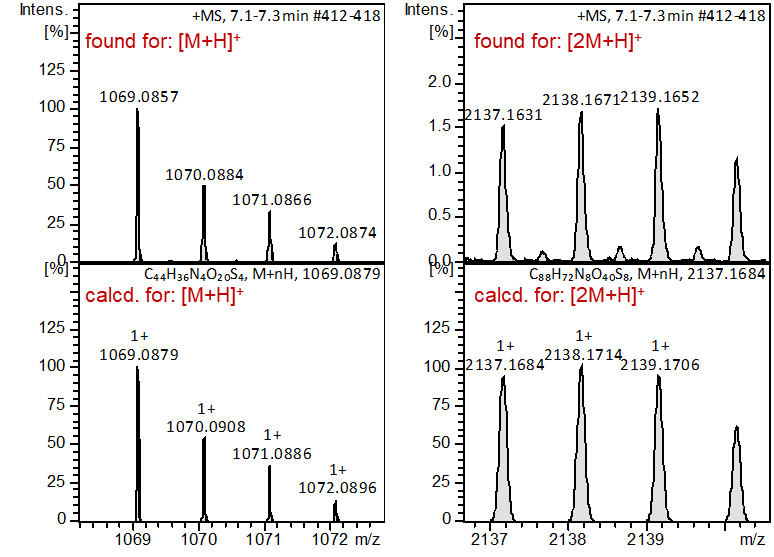


**Figure S20:** LC-MS analysis of **A_2_-4Hyd**. Extracted ion chromatogram, +ESI-MS spectrum, simulated/found spectra comparison.

**A_3_**


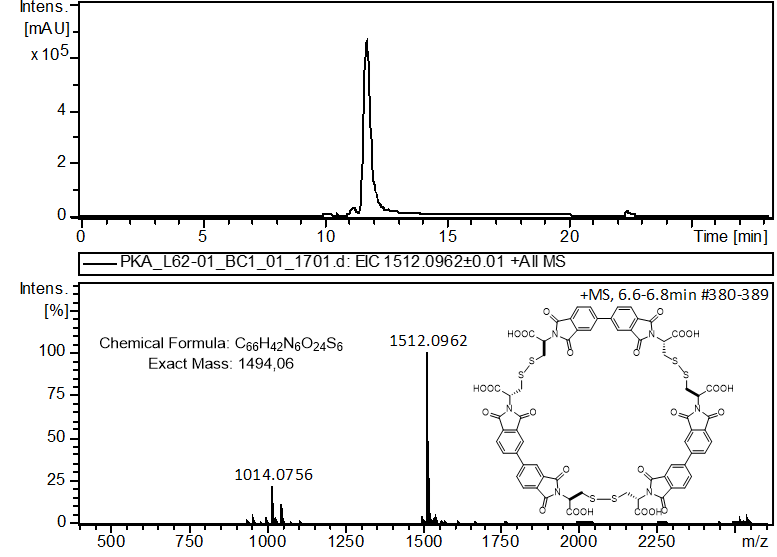


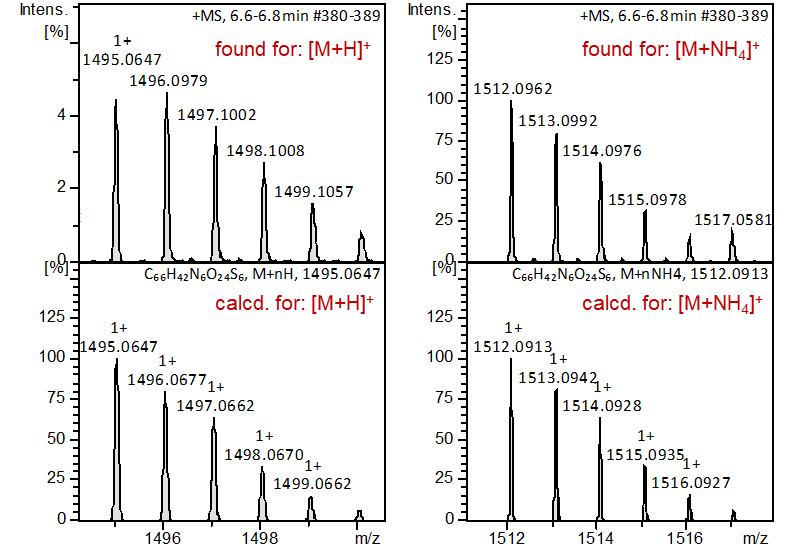


**Figure S21:** LC-MS analysis of **A_3_**. Extracted ion chromatogram, +ESI-MS spectrum, simulated/found spectra comparison.

**A_3_-Hyd**


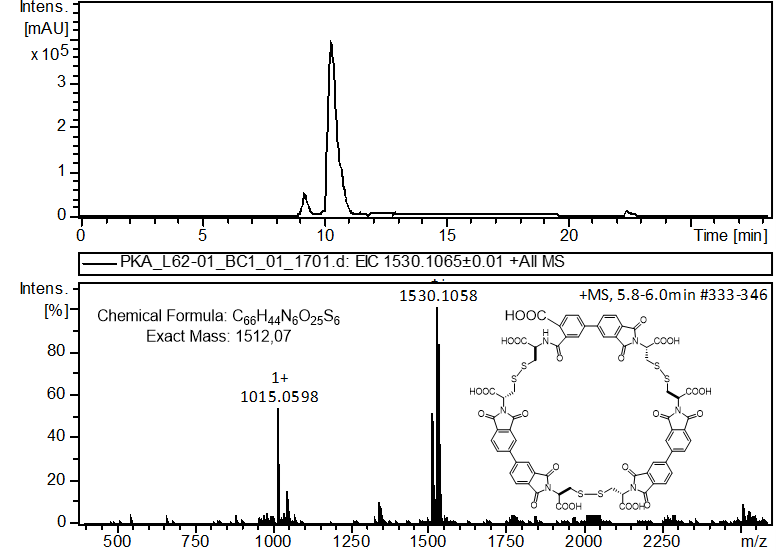


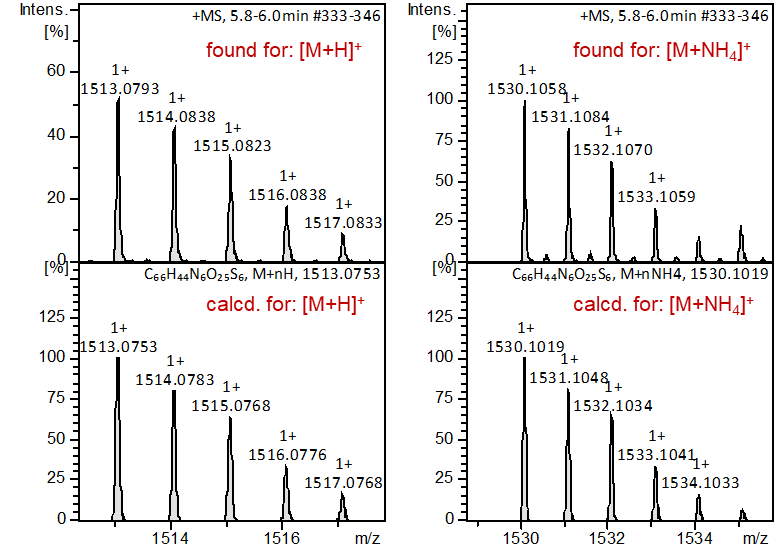


**Figure S22:** LC-MS analysis of **A_3_-Hyd**. Extracted ion chromatogram, +ESI-MS spectrum, simulated/found spectra comparison.

**A_4_**


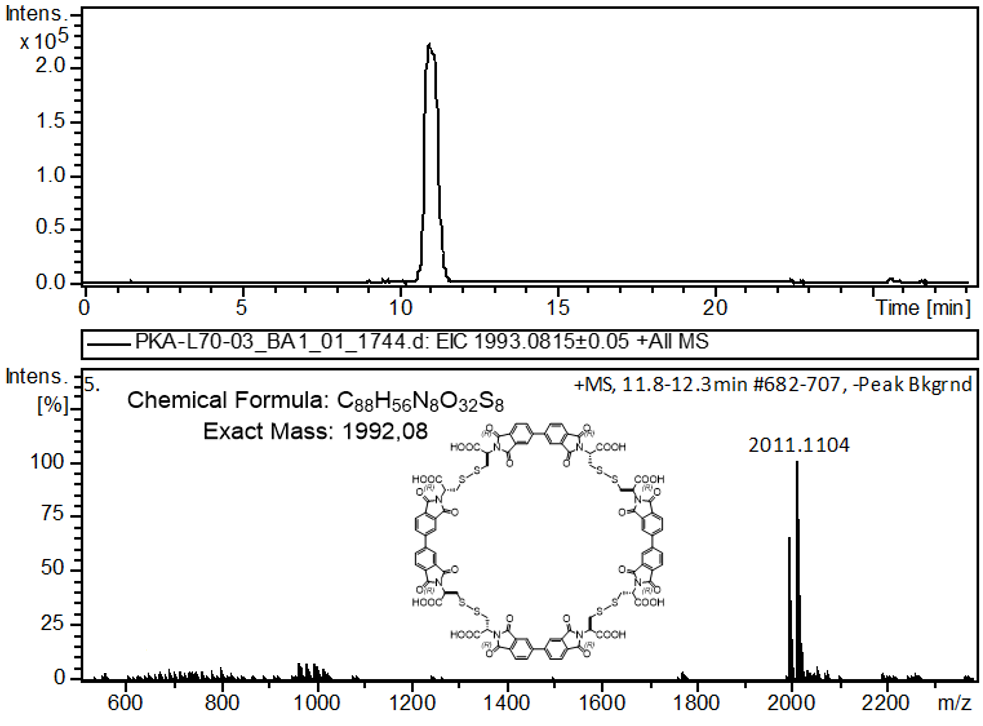


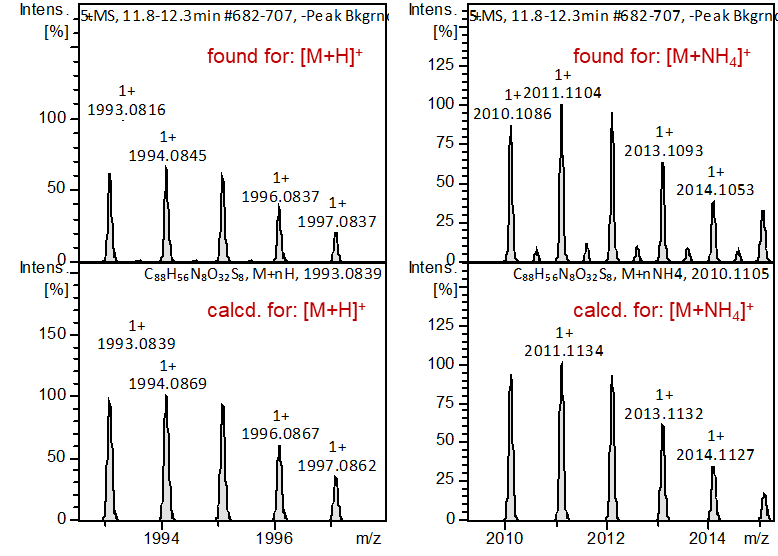


**Figure S23:** LC-MS analysis of **A_4_**. Extracted ion chromatogram, +ESI-MS spectrum, simulated/found spectra comparison.

**A_4_-Hyd**


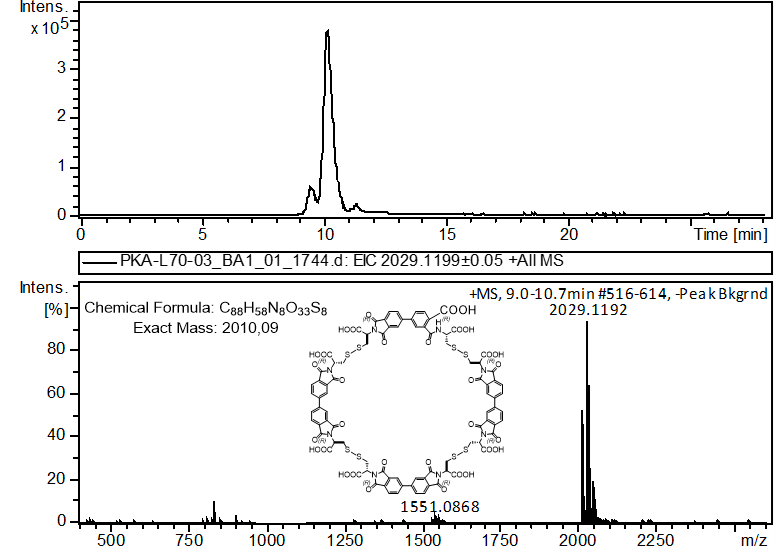


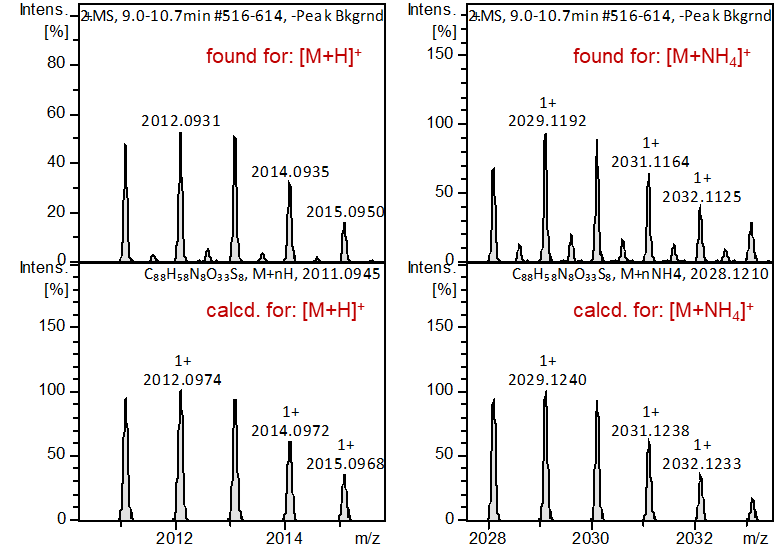


**Figure S24:** LC-MS analysis of **A_4_-Hyd**. Extracted ion chromatogram, +ESI-MS spectrum, simulated/found spectra comparison.

**AS**


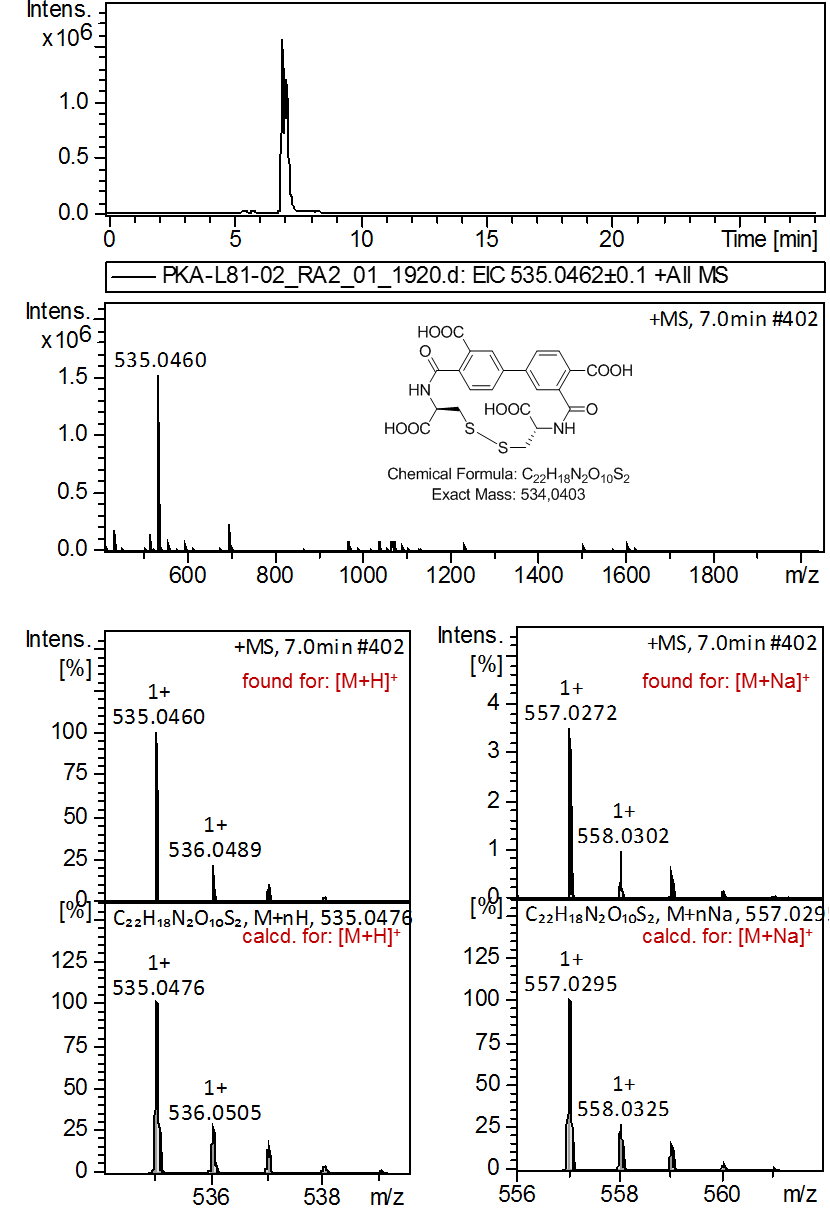


**Figure S25:** LC-MS analysis of **AS**. Extracted ion chromatogram, +ESI-MS spectrum, simulated/found spectra comparison.

**AA**


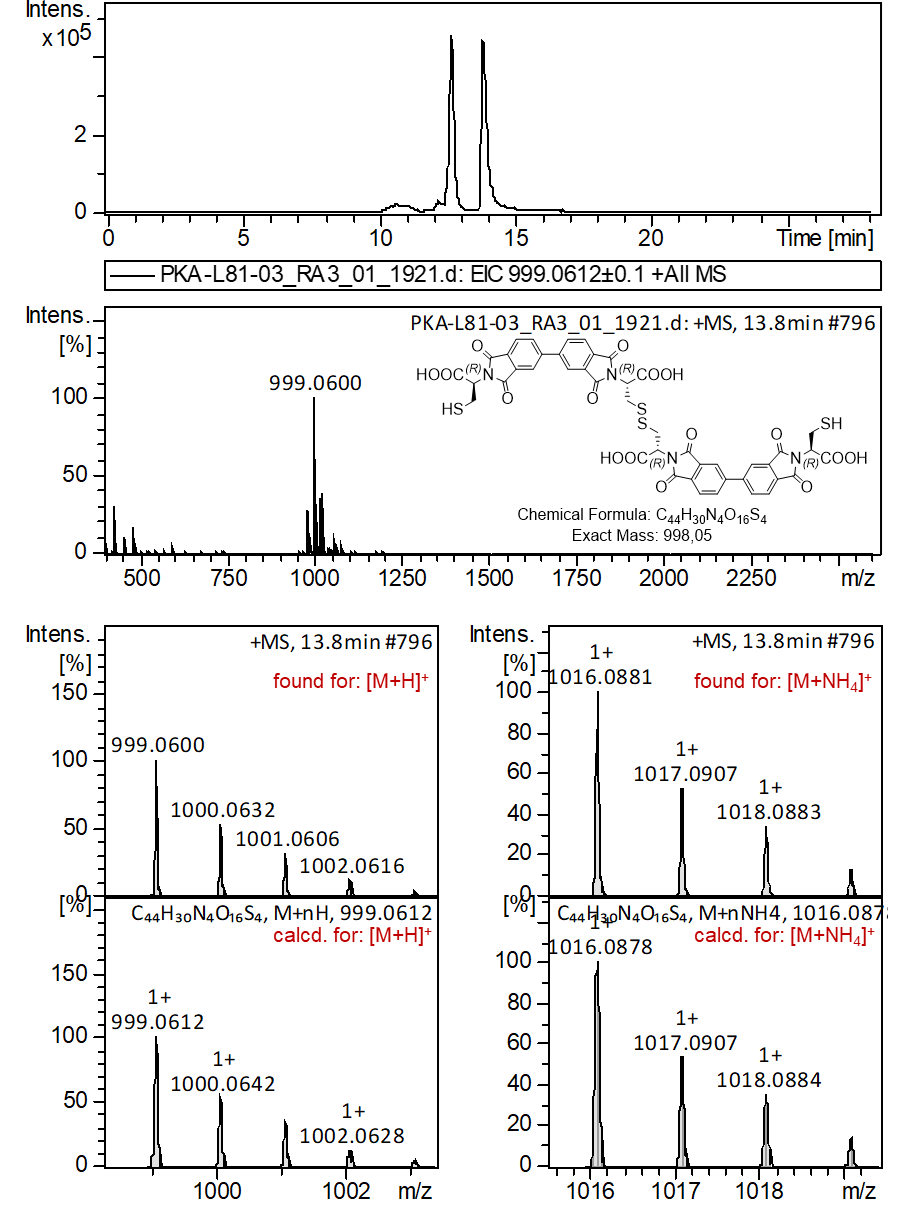


**Figure S26:** LC-MS analysis of **AA**. Extracted ion chromatogram, +ESI-MS spectrum, simulated/found spectra comparison.

**AA-Hyd**


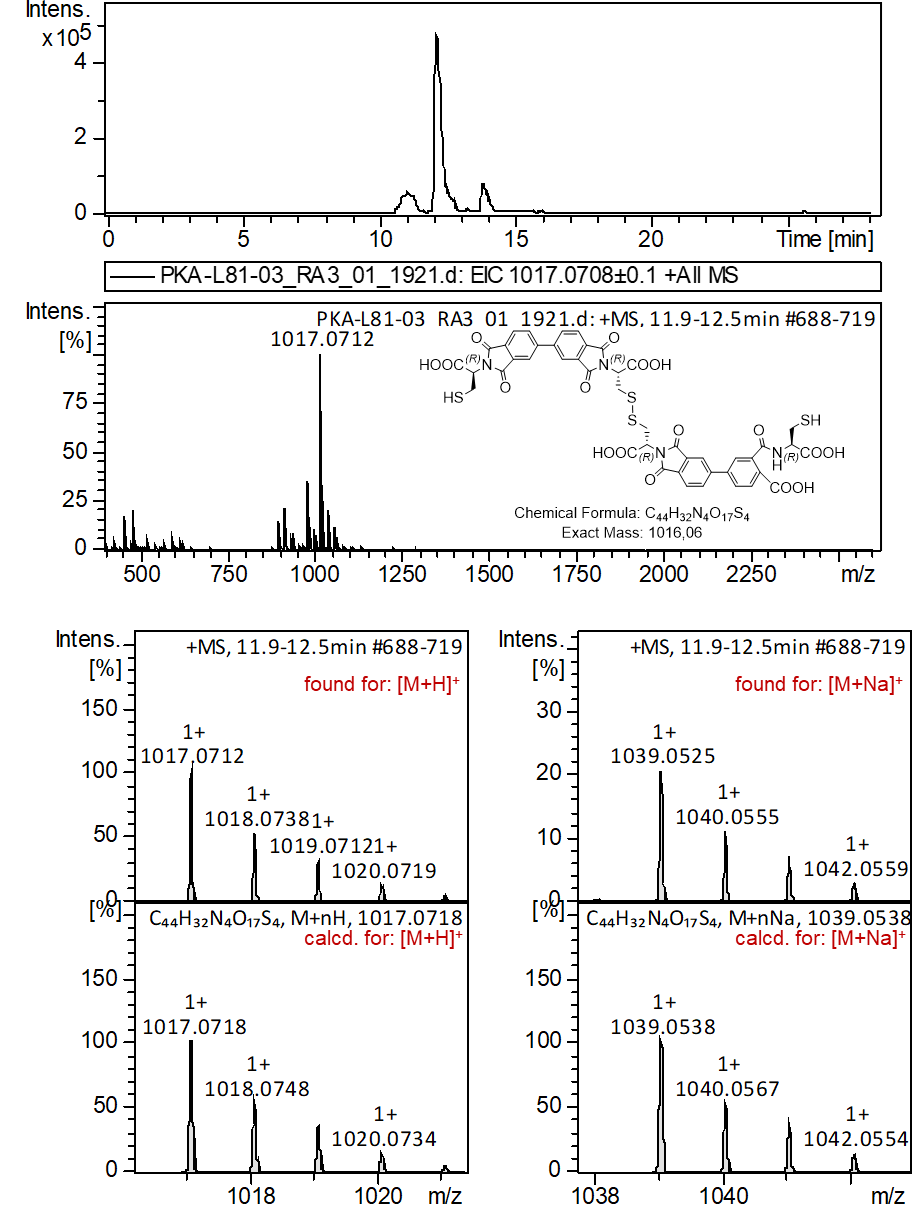


**Figure S27:** LC-MS analysis of **AA-Hyd**. Extracted ion chromatogram, +ESI-MS spectrum, simulated/found spectra comparison.

# HPLC Data

**DMSO/Water optimization**


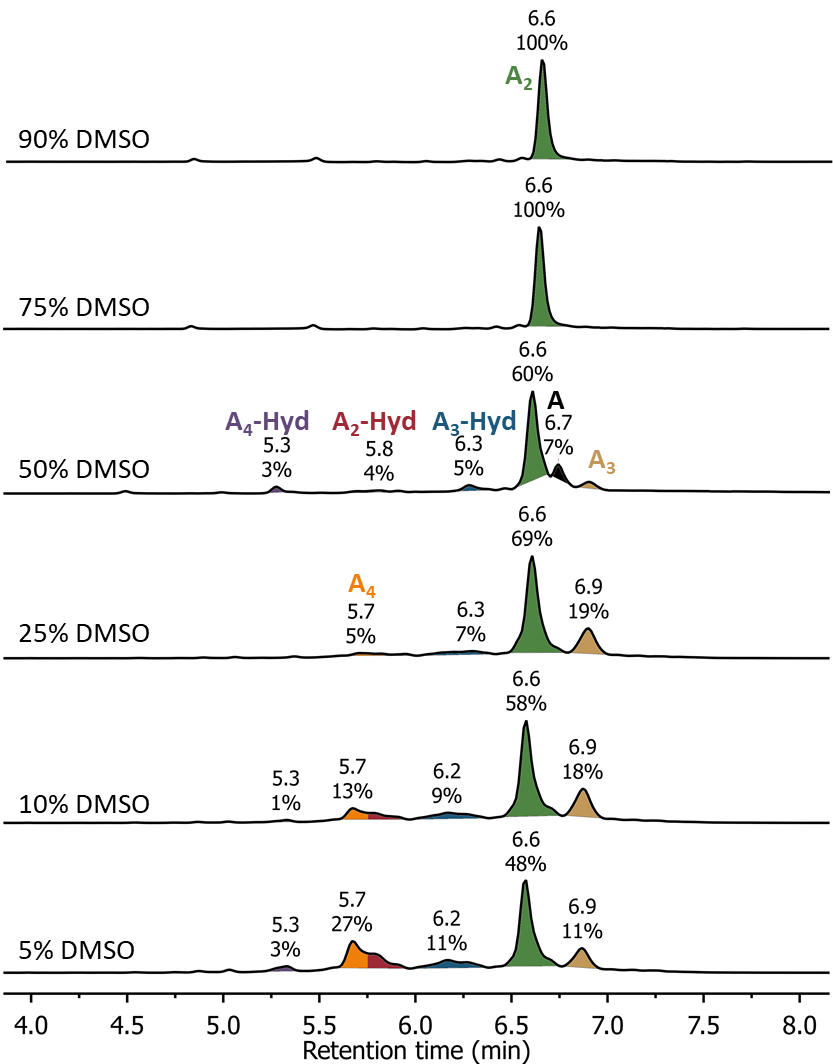


**Figure S28:** HPLC traces (254 nm) of DMSO/Water optimization DCLs.

**DMSO/0.1M AcONH_4_ optimization**


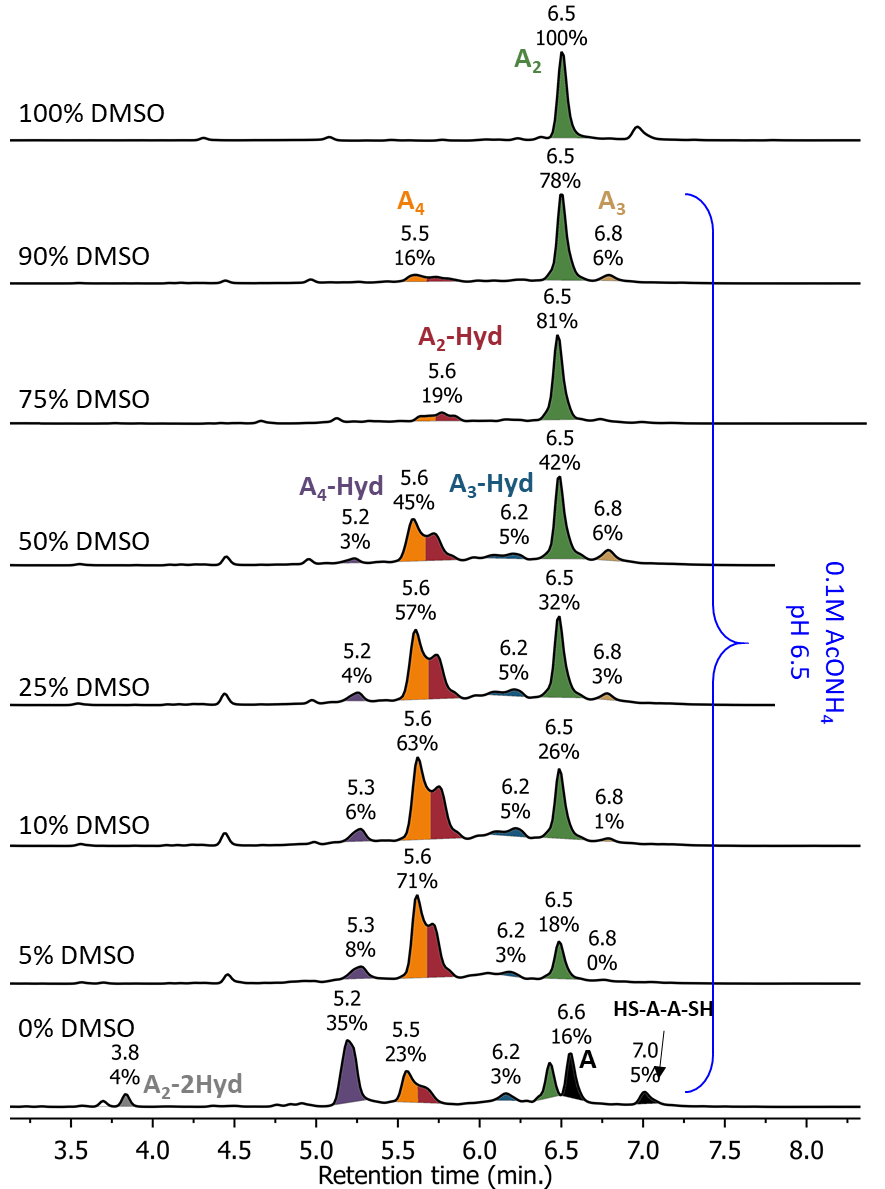


**Figure S29:** HPLC traces (254 nm) of DMSO/0.1M AcONH_4_ optimization DCLs.

**pH in 5%DMSO/0.1M AcONH_4_ optimization**


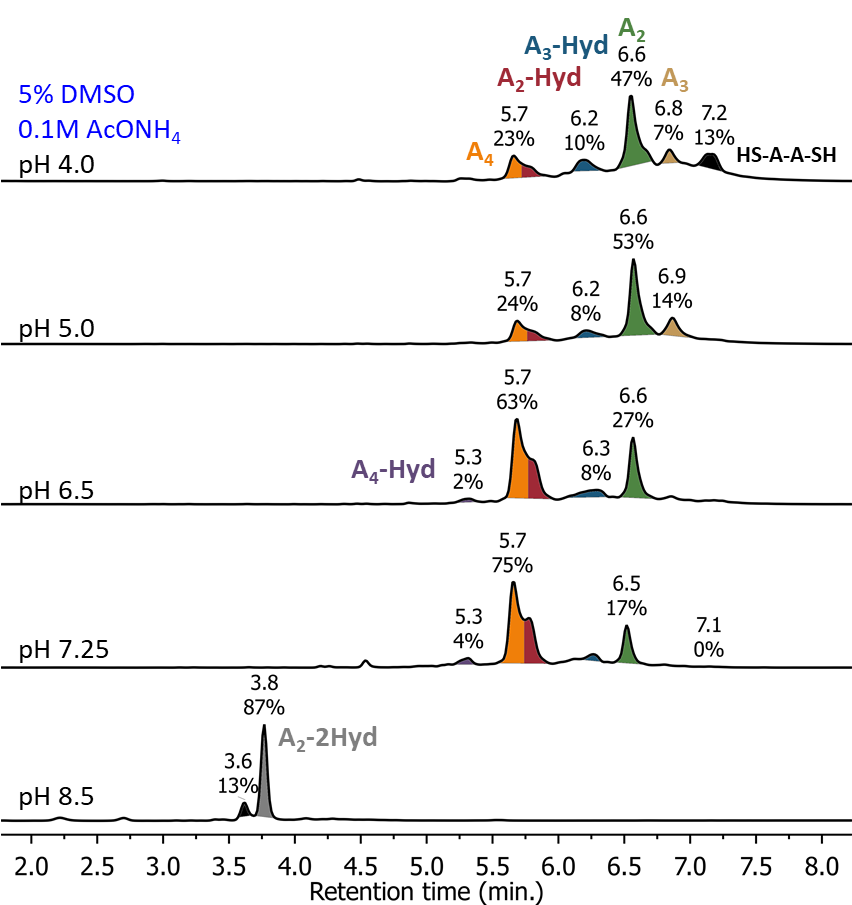


**Figure S30:** HPLC traces (254 nm) of pH in 5% DMSO/0.1M AcONH_4_ optimization DCLs.

**Figure S31:** Scheme of the N-phthalimide derivative hydrolysis mechanism in basic conditions.^[3]^


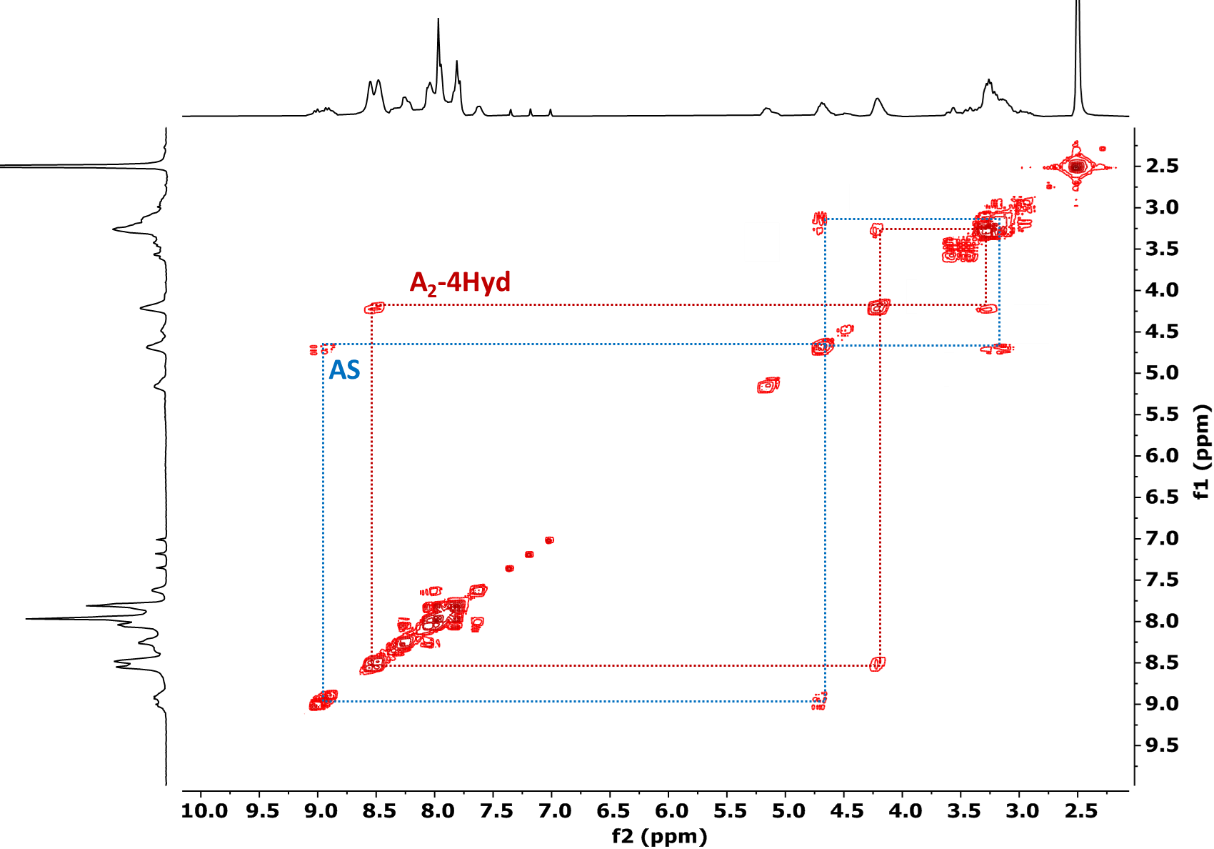


**Figure S32:** COSY NMR spectrum of **A_2_-4Hyd** in DMSO-*d_6_* at 298 K (300 MHz).


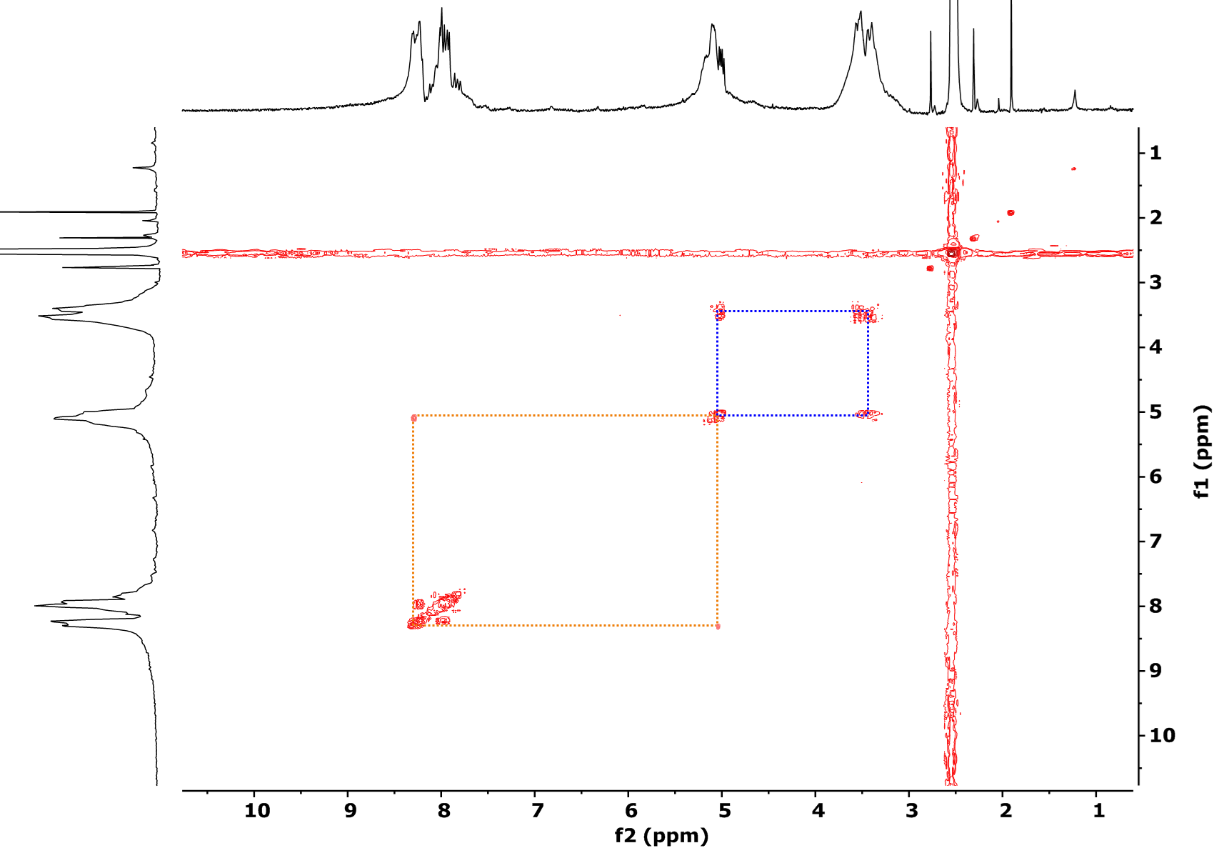


**Figure S33:** COSY NMR spectrum of **A** DCL (pH 7.25, 5% DMSO, Fig. 3f) in DMSO-*d_6_* at 298 K (300 MHz).

**
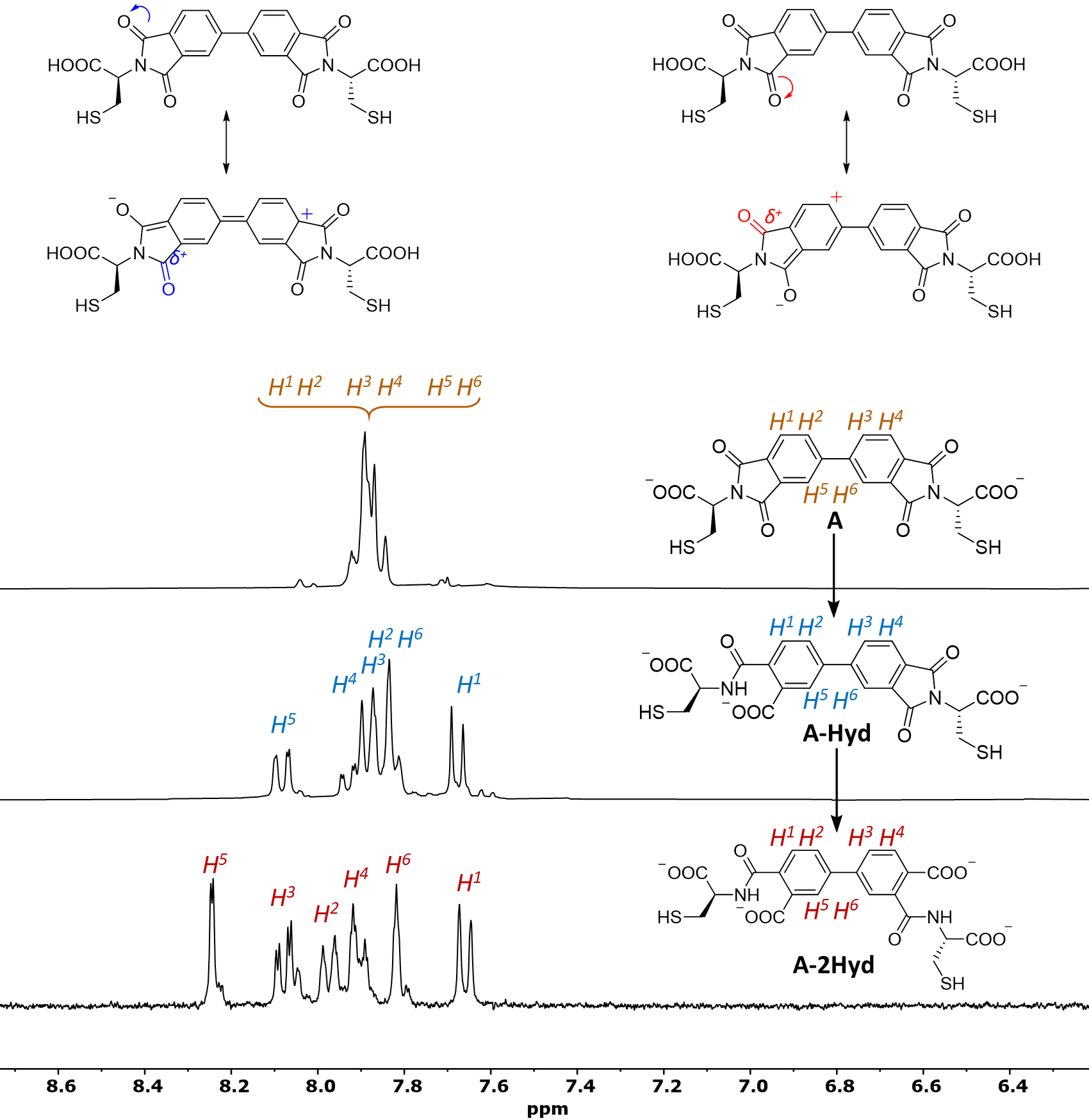
**

**
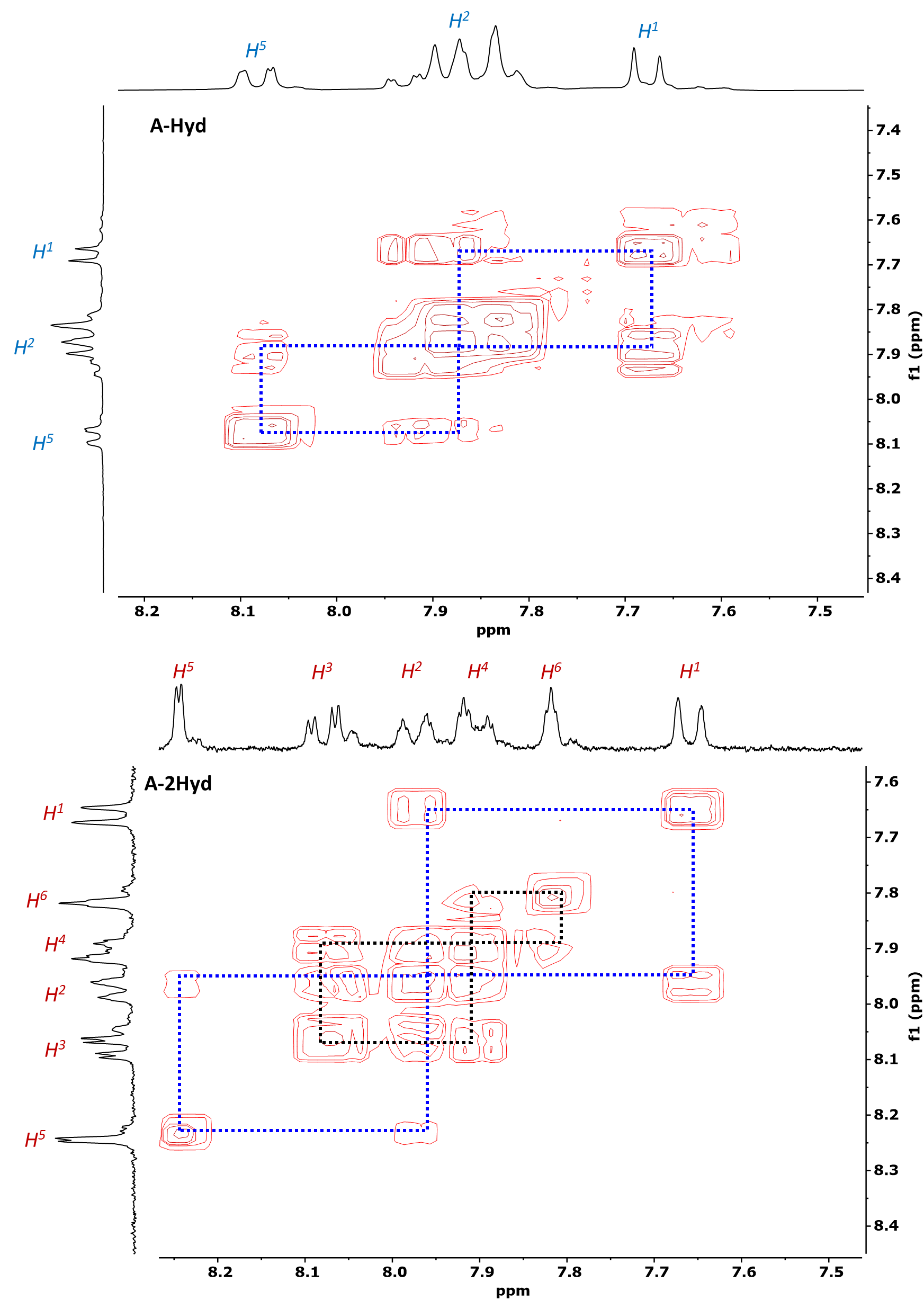
**

**Figure S34:** Comparison of ^1^H NMR and COSY spectra of **A** to **A-2Hyd** hydrolysis in time (D_2_O at 298 K, 300 MHz).

Four imide carbonyls of **A** can act as electron withdrawing groups resulting in the accumulation of single positive charge on the phenyl ring. A more stable resonance structure (due to formation of tertiary carbocation) is generated when electron withdraw takes place on the O-carbonyl atom in the *para* position with respect to the single bond between aromatic rings (see ESI, Fig. S34, left path). This makes the second carbonyl group (in the *meta* position, marked blue) more electrophilic, thus directing the hydrolysis process in this position. This has been confirmed by the ^1^H NMR observation of this hydrolysis over time, which showed that the *para* amide was formed first (see Fig. S34).^[4]^


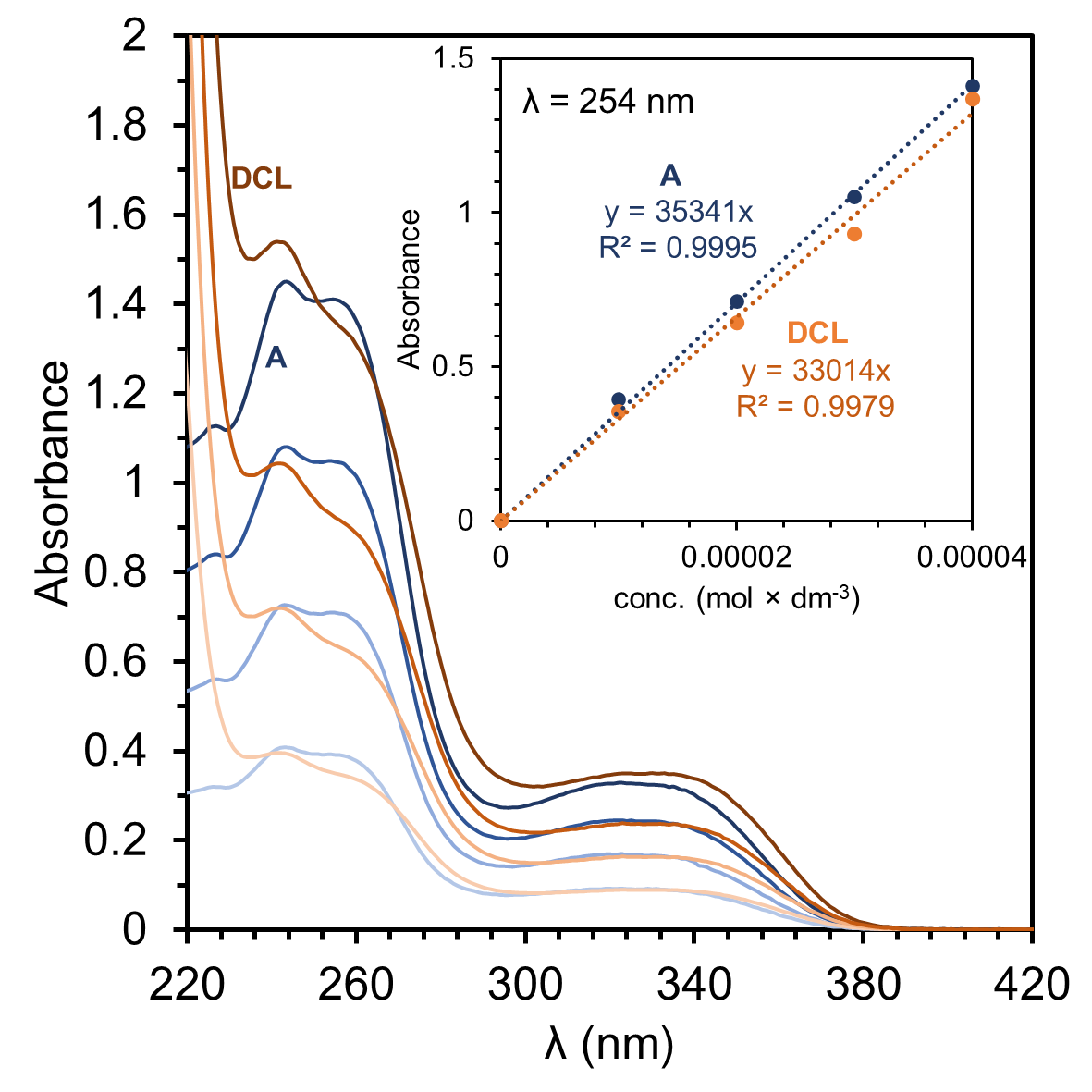


**Figure S35**: Comparison of UV-Vis spectra of **A** (blue) and DCL (brown, pH 6.5, conc. 1x10^-5^ M – 4x10^-5^ M, cuvette 1×1 cm). Inset contains the comparison of absorbance values at 254 nm at the same concentrations.

# References

[1] M. Konopka, G. Markiewicz, A. R. Stefankiewicz, *RSC Adv.* **2018**, *8*, 29840-29846.

[2] A. R. Stefankiewicz, M. R. Sambrook, J. K. M. Sanders, *Chem. Sci.* **2012**, *3*, 2326.

[3] M. N. Khan, A. A. Khan, *J. Chem. Soc. Perkin Trans. II* **1979**, *-*, 796-798.

[4] P. L. Corio, B. P. Dailey, *J. Am. Chem. Soc.* **1956**, *78*, 3043-3048.
